# The colonization of land was a likely driving force for the evolution of mitochondrial retrograde signalling in plants

**DOI:** 10.1093/jxb/erac351

**Published:** 2022-09-03

**Authors:** Kasim Khan, Olivier Van Aken

**Affiliations:** Department of Biology, Lund University, Lund, Sweden; Department of Biology, Lund University, Lund, Sweden; University of Glasgow, UK

**Keywords:** Chloroplasts, colonization of land, evolution, mitochondria, plants, retrograde signalling, stress response

## Abstract

Most retrograde signalling research in plants was performed using Arabidopsis, so an evolutionary perspective on mitochondrial retrograde regulation (MRR) is largely missing. Here, we used phylogenetics to track the evolutionary origins of factors involved in plant MRR. In all cases, the gene families can be traced to ancestral green algae or earlier. However, the specific subfamilies containing factors involved in plant MRR in many cases arose during the transition to land. NAC transcription factors with C-terminal transmembrane domains, as observed in the key regulator ANAC017, can first be observed in non-vascular mosses, and close homologs to *ANAC017* can be found in seed plants. Cyclin-dependent kinases (CDKs) are common to eukaryotes, but E-type CDKs that control MRR also diverged in conjunction with plant colonization of land. *AtWRKY15* can be traced to the earliest land plants, while *AtWRKY40* only arose in angiosperms and *AtWRKY63* even more recently in Brassicaceae. Apetala 2 (AP2) transcription factors are traceable to algae, but the ABI4 type again only appeared in seed plants. This strongly suggests that the transition to land was a major driver for developing plant MRR pathways, while additional fine-tuning events have appeared in seed plants or later. Finally, we discuss how MRR may have contributed to meeting the specific challenges that early land plants faced during terrestrialization.

## Teaser

Phylogenetic analysis shows that the transition to land was a major driving force for developing mitochondrial retrograde pathways in plants, while additional fine-tuning appeared in seed plants or even later.

## Introduction

Around 500 million years ago (MYA), the appearance of plants on land, which probably evolved from freshwater unicellular algae, is one of the most significant evolutionary developments in Earth’s history. The event had a major impact on the biosphere in terms of increasing atmospheric oxygen levels, paving the way for the development of a terrestrial ecosystem on the planet (Delwiche and Cooper, 2015). The transition had its many challenges for the land plants, forcing them to evolve again and again through key innovations, for instance evolution of specialized cells, tissues, and organs, to enhance their survival chances and reproduction (Bowman, 2013). Mitochondria are essential double-membrane-bound subcellular compartments that are often regarded as the cell’s ‘powerhouse’, since the majority of the energy required to maintain cellular activities is provided by mitochondria through aerobic respiration in the form of ATP; however, in green plants, chloroplasts and mitochondria work in tight coordination to provide cellular energy (Chadee *et al.*, 2021). At present we have sufficient evidence to believe that eukaryotes are the product of a fusion between an aerobic prokaryotic cell, a relative of modern α-proteobacteria, and a host archaeon (Martijn *et al.*, 2018). The theory was later recognized as ‘endosymbiotic theory’. Over time, most of the genetic information encoded by endosymbiotic mitochondria was transferred to the host cell nucleus. As a result, eukaryotic cells have gained more ability to modulate mitochondrial functions by regulating the flow of mitochondrial proteins (anterograde signals) and responding to mitochondrial dysfunction. Furthermore, mitochondria acquired the capacity to relay signals to other organelles to build a feedback communication system with the nucleus to communicate information on its functional status (Welchen *et al.*, 2014). This feedback mechanism is known as mitochondrial retrograde regulation (MRR). Thus, mitochondria act as cellular sensors to environmental perturbation when mitochondrial metabolic pathways are disrupted in response to a wide range of abiotic stresses such as drought, cold, excessive light, hypoxia, salinity, UV-B, etc., as well as various biotic stresses (Bohovych and Khalimonchuk, 2016; Welchen and Gonzalez, 2016).

In eukaryotes, mitochondrial dysfunction caused by environmental stress triggers mitochondrial retrograde signalling pathways that regulate the expression of nuclear genes encoding mitochondrial proteins (NGEMPs) and cellular metabolism to divert energy from growth and developmental processes to stress responses (Ivanova *et al.*, 2014; Kerchev *et al.*, 2014; Crawford *et al.*, 2018). During colonization of land by plants, the streptophyte algal ancestors faced many new challenges including drought, heat, excess light stress, altered atmospheric oxygen levels, and pathogens. To meet these challenges, the cell requires an effective sensor system to maintain homeostasis and plant growth. In this manuscript, we aim to trace the evolutionary origins of the factors currently known to be involved in MRR in land plants (Viridiplantae) and discuss the evolutionary significance of several genetic and physiological components of retrograde signalling. Since a series of studies confirmed the critical role of retrograde signalling in plant responses to a wide range of environmental stimuli, we further explore how these regulators of MRR helped in survival of early land plants in the challenging climatic conditions during their movement to land.

In general, retrograde signalling is triggered by a mitochondrial signal generated as a result of mitochondrial perturbations and reaching the nucleus via interactions with other cellular compartments. Although the principle of mitochondrial retrograde signalling is conserved throughout the evolution of eukaryotes, with examples known from single-cell yeasts to mammals and angiosperms, the same is not true for the molecular regulators of lineage-specific retrograde pathways (Tran and Van Aken, 2020). Yeast (*Saccharomyces cerevisiae*) is a model system widely studied to understand mitochondrial evolution and hosts the first extensively characterized ‘retrograde’ (RTG)-based pathway that includes cytosolic transcriptional activators, Rtg1p and Rtg3p (Parikh *et al.*, 1987; Liu and Butow, 2006). Rtg1p and Rtg3p belong to the basic helix–loop–helix/leucine zipper (bHLH/LeuZip) transcription factor (TF) family, together forming a heterodimer activator that translocates from the cytosol to the nucleus to control the expression of genes encoding mitochondrial proteins (Sekito *et al.*, 2000). A drop in intracellular ATP levels during mitochondrial dysfunction allows Rtg2p, an activator of the pathway, to trigger translocation of Rtg1/3p to the nucleus (Sekito *et al.*, 2002; Liu *et al.*, 2003). Similarly, triggers of different mitochondrial retrograde signalling pathways have been identified in different animal systems. Disruption of the mitochondrial membrane potential is shown to be a principal cause of MRR in mammalian cells, affecting mitochondrial Ca^2+^ absorption and an increase in free Ca^2+^ in the cytoplasm. The increased cytosolic Ca^2+^ activates ATF2 (activating transcription factor 2) translocation to the nucleus to start transcriptional responses. Other pathways, for example nuclear factor of activated T-cells (NF-AT) and NF-κB, also mediate retrograde responses in mammalian cells (Biswas *et al.*, 1999, 2003). Interestingly, none of the regulatory genes involved in yeast or animal MRR seem to be conserved in plants, indicating that MRR has evolved in different directions across kingdoms (Tran and Van Aken, 2020).

In plants, MRR is widely studied in *Arabidopsis thaliana*, which is often monitored by the induction of alternative oxidase *AOX1a* transcripts in response to mitochondrial stress (Vanlerberghe and McIntosh, 1992; Djajanegara *et al.*, 2002; Wang *et al.*, 2018; Qiao *et al.*, 2022). *AOX1a* is up-regulated by different environmental stress conditions including nutrient deficiency, temperature, desiccation, oxygen availability, and pathogen infection, and also by several pharmacological compounds causing mitochondrial perturbations such as inhibitor of Complex III and antimycin A (Cvetkovska and Vanlerberghe, 2012a; Wang *et al.*, 2018). Based on expression levels of *AOX1a* and other Arabidopsis MRR target genes, several factors involved in MRR were identified by forward and reverse genetics approaches. Major MRR signalling components identified in plants so far include ANAC017-related, ABI4, and WRKY TFs, as well as the cyclin-dependent kinase CDKE;1, as reviewed by Ng *et al.* (2014).

Here, we aimed to answer the question of how and when these regulators and pathways involved in MRR appeared during land plant evolution. We also wanted to explore to what extent the transition to land was a driving force to develop retrograde signalling pathways, helping early land plants to adapt to the new environment. This analysis will help us evaluate if MRR signalling components could have been pre-adaptive factors to allow the development of plant terrestrialization, or whether they were developed concurrently with or after land transition. Therefore, we tracked the evolution of regulators of retrograde signalling in ancestral plants by using genomic and proteomic data.

## Materials and methods

### Phylogenetic analysis

The putative ABI4 protein sequences representing significant evolutionary groupings were used to create an ABI4 alignment. Putative ABI4 protein sequences retrieved from Phytozome (https://phytozome-next.jgi.doe.gov/) and the NCBI Gene Bank were aligned by the online MAFFT sequence alignment server (https://mafft.cbrc.jp/alignment/server/) with default parameters (Katoh *et al.*, 2019). ABI4-specific conserved domains have been found based on previous studies (Wind *et al.*, 2013; Gregorio *et al.*, 2014).

For CDK phylogenetic analysis, CDK family protein sequences were obtained by Blast searches using Arabidopsis CDKs as queries against the NCBI non-redundant protein database (http://www.ncbi.nlm.nih.gov/), while human CDK8 was obtained from the NCBI gene bank. After filtering out duplicated sequences, full-length putative CDK proteins from representative plants were aligned by MAFFT and the phylogenetic ML tree was constructed by using IQ-TREE with 1000 bootstraps (Trifinopoulos *et al.*, 2016). Similarly, a phylogenetic tree of CDKEs was constructed by using plant CDKE and human CDK8 proteins. Phylogenetic analysis of the WRKY family was largely based on the data provided by Mohanta *et al.* (2016).

Proteins sequences for all NAC (NAM, ATAF, and CUC) genes from selected species covering land plants were collected from PLAZA 4.0 (Van Bel *et al.*, 2018). NAC sequences from *Klebsormidium nitens* (previously known as *K. flaccidum*) were collected from the Plant Transcription Factor Database (http://planttfdb.gao-lab.org/; Jin *et al.*, 2017). All collected NAC family protein sequences were aligned using MAFFT (Katoh *et al.*, 2019) and edited for gaps prior to phylogenetic tree construction with IQ-TREE using 1000 bootstraps. Transmembrane domain prediction was performed using TM-HMM (https://services.healthtech.dtu.dk/service.php?TMHMM-2.0) (Krogh *et al.*, 2001). For the rooted tree focusing on the ANAC017 subgroup (see Fig. 3B below), we further edited the sequences focusing on the NAC domain-containing N-terminus itself to avoid gaps introduced by NAC analogues with only distant similarity to Arabidopsis ANAC017. The rooted tree was inferred using iqTREE (Trifinopoulos *et al.*, 2016). For further phylogenetic analysis of transmembrane domain-containing ANAC017-like proteins in plants in a broad sense, NAC sequences from different groups of Archaeplastida including algae, bryophytes, lycophytes, ferns, gymnosperms, and angiosperms from the One KP database (One Thousand Plant Transcriptomes Initiative, 2019) were obtained by using ANAC017 as query sequence in the protein blast tool. The 10 000 best hits for ANAC017 in the One KP set were retrieved, and the highest ranking hit for each species was retained, resulting in 880 proteins from unique species. The sequences of the 880 first-ranked BLAST hit for the different plant species, along with the 94 Arabidopsis NACs obtained from PLAZA, were aligned by MAFFT, and a phylogenetic tree was constructed by IQ-tree with 1000 bootstraps.

Phylogenetic trees were visualized using Figtree v1.4.3. All the raw files generated from MAFFT alignment and IQ-TREE are available in Supplementary Dataset S1. The IQ-TREE log files for each phylogenetic tree provides the run information and parameters used in the study, whereas original alignment and tree files are also available in the Supplementary Dataset S1.

## Results

### The evolutionary perspective of major plant MRR regulators

#### Abscisic acid-insensitive 4 (ABI4)

**Fig. 1.**
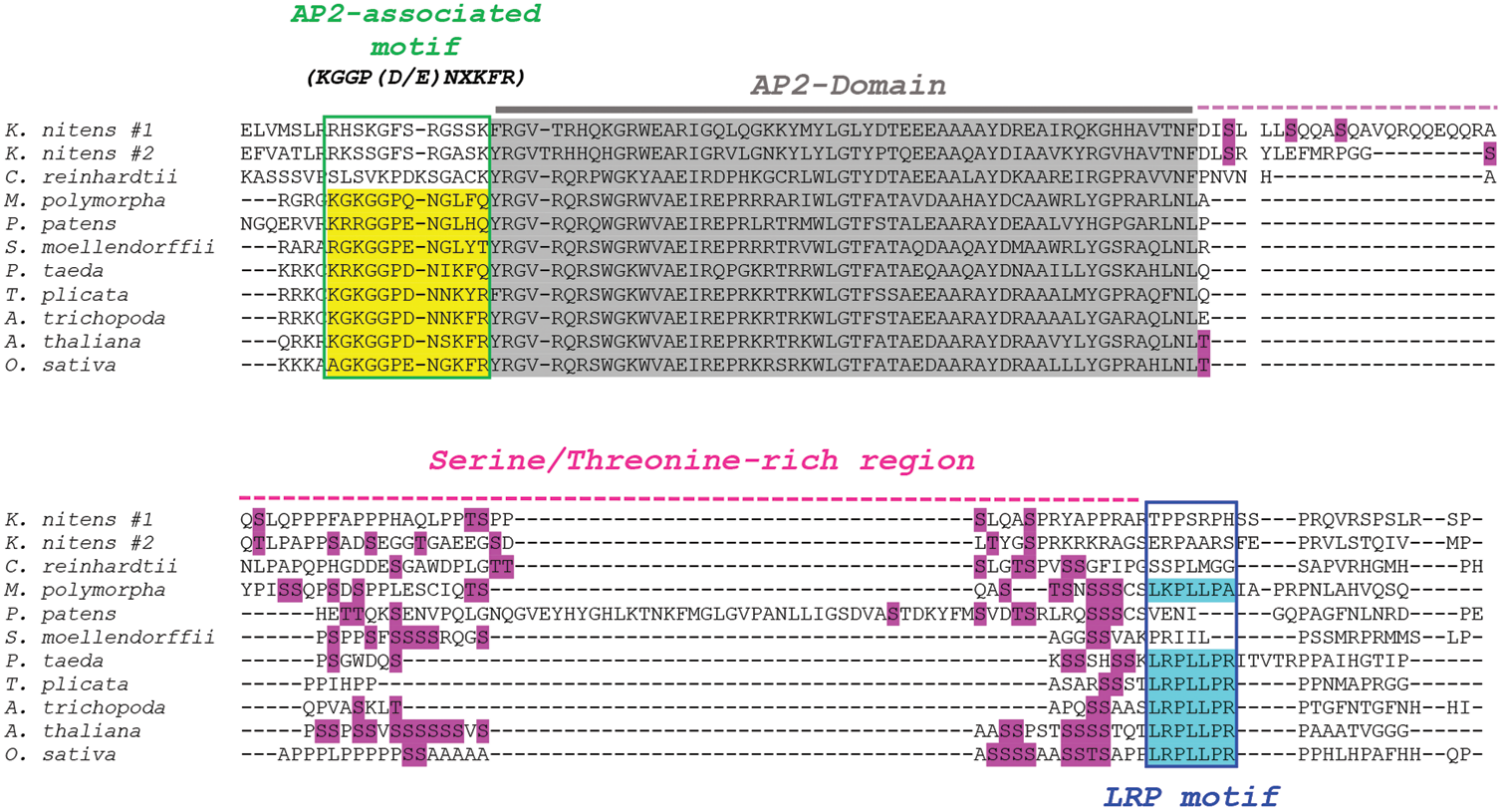
ABI4-specific signatures in representative streptophyte plants. Protein sequences of Arabidopsis ABI4 homologues representing different evolutionary groups were retrieved from Phytozome and the NCBI Gene Bank. The obtained sequences were aligned using the online MAFFT sequence alignment server. ABI4-specific conserved domains, AP2-associated motif (yellow), the AP2 domain (grey), the serine/threonine-rich region (pink), and the LRP motif (cyan) were identified and indicated in the mentioned colour schemes.

ABI4 was the first transcription factor (TF) shown to affect the expression of *AOX1a* by acting as a suppressor and regulating genes encoding both mitochondrial and chloroplast proteins (Koussevitzky, 2007; Giraud *et al.*, 2009), though the role of *ABI4* in chloroplast retrograde signalling has been questioned (Kacprzak *et al.*, 2019). ABI4 belongs to the AP2/ERF gene family of plant-specific TFs and is characterized by the presence of the APETALA 2 (AP2) DNA-binding domain (Shigyo *et al.*, 2006). *ABI4* is the only member of the DREB subfamily’s A3 subgroup in *A. thaliana*. In addition to the conserved AP2 domain, other conserved motifs were also identified in ABI4 orthologues from angiosperms, including an AP2-associated domain and the highly conserved ‘ABI4’ or ‘LRP’ motif (LRPLLPRP). The AP2-associated motif is closely related to DREB TFs of the A2 subgroup of the AP2/ERF family and required to provide protein stability and subcellular localization motifs (Wind *et al.*, 2013; Gregorio *et al.*, 2014). Notably, these unique identified motifs are specific to the ABI4 gene and not present in other subgroups of the AP2/ERF gene family. We carefully searched for the presence of ABI4 in evolutionarily distinct groups of plants. Green algae such as *Klebsormidium nitens* and *Chlamydomonas reinhardtii* contained genes with clear AP2 domains, but we did not find any match for the ABI4-unique AP2-associated and LRPLLPRP (LRP) motifs (Fig. 1; Supplementary Fig. S1). Notably, *K. nitens* is a multicellular charophytic alga and thought to be related to the ancestors of present-day land plants. Further analysis of land plants showed that the AP2-associated motif was found in all examined land plants, while the LRPLLPRP ‘ABI4’ motif was only consistently found in seed plants (gymnosperms and angiosperms). In agreement, Wind *et al.* (2013) and Gregorio *et al.* (2014) individually suggested that the ‘ABI4’ motif (LRPLLPRP) is conserved in angiosperms as well as in the gymnosperm *Pinus taeda*, while ABI4 orthologues appear to be absent from ancestral algae and mosses. We searched the genome of additional lineages and could indeed confirm a perfectly conserved ‘ABI4’ motif in the angiosperm sister group member *Amborella trichopoda* and gymnosperms *Pinus taeda* and *Thuja plicata*, but lycophytes and non-vascular plants did not consistently appear to have AP2 domain proteins with the ABI4 motif. Interestingly, the liverwort *Marchantia polymorpha* contained an LKPLLPA motif at the same position in the alignment, which is highly similar to the LRPLLPR motif observed in the seed plants. Some previous reports also suggested the presence of ABI4 orthologues (ABI4-like proteins) in algae and basal land plants, but, according to our analysis, this is clearly not the case in *Physcomitrium patens* or *Selaginella moellendorffii* (Mizoi *et al.*, 2012; Wind *et al.*, 2013). A recent report also suggested an ABI4-like gene in the desert moss *Bryum argenteum* (*TR118609_c0_g1_i1*; QDB64618.1). This gene indeed contains the AP2-associated motif, but again lacks the LRPLLPR motif found in ABI4 in seed plants (Li *et al.*, 2018). It is important to note that abscisic acid (ABA) responses are already known in ancestral algae, and ABA signalling might be a key component of retrograde signalling enabling ancestral plants to sense altered climatic conditions during terrestrialization (Bajguz, 2009; Holzinger *et al.*, 2014; Hori *et al.*, 2014; Zhao *et al.*, 2019). Therefore, ABI4 is likely to have evolved in its typical (Arabidopsis-like) form relatively late with the appearance of seed plants, though the AP2 family can be traced back to microalgae (Thiriet-Rupert *et al.*, 2016). As the AP2-associated domain seems to be conserved across land plants but not green algae, and some bryophytes already contain an ABI4-like motif, it seems that the evolutionary pressure that led to ABI4 development probably appeared in conjunction with the transition to land.

#### WRKY transcription factors

Several WRKY TFs were identified to regulate nuclear-encoded mitochondrial proteins. *AtWRKY40* was found to be a repressor of MRR genes such as *AOX1a* and *UPOX* during high-light stress and antimycin A-induced mitochondrial dysfunction, while *AtWRKY63* acts as an activator (Van Aken *et al.*, 2013). *AtWRKY40* and *AtWRKY63* are particularly involved in regulating the expression of genes responding commonly to both mitochondrial and chloroplast dysfunction, but not of genes responding to either mitochondrial or chloroplast perturbation (Van Aken *et al.*, 2013). Overexpression of *AtWRKY15* led to inhibition of mitochondrial stress responses in Arabidopsis during salt stress (Vanderauwera *et al.*, 2012). The expression of *ABI4* is tightly controlled by WRKY18, WRKY40, and WRKY60, which may provide another potential feedback loop (Liu *et al.*, 2012). WRKY TF genes evolved and amplified during the course of evolution from aquatic green algae to modern land plants (Chen *et al.*, 2019). In addition to plants, WRKY genes are also traced to other eukaryotic non-plant lineages including some diplomonads, amoebae, and fungi, probably originating from multiple lateral gene transfer events (Zhang and Wang, 2005). The WRKY gene family is mainly characterized by the presence of an ~60 amino acid long WRKY DNA-binding domain (WRKY DBD with the WRKYGQK core motif) and zinc-finger motifs, and is divided into three groups: Group I (two WRKY DBDs), Group II (a single DBD with a C2H2 zinc finger), and Group III (a single DBD with a different C2HC zinc finger) (Rushton *et al.*, 2010). WRKY Group I is only present in unicellular algae and apparently has no counterpart in land plants. However, detailed analyses in *K. nitens* suggested the presence of Group IIb WRKY genes, which also have hallmarks of Group IIb genes from mosses and flowering plants (Rinerson *et al.*, 2015). Further diversification and expansion of the WRKY gene family seems to have occured in early land plants such as mosses (*P. patens*) and spike moss (*S. moellendorffii*), and can be observed in current-day vascular plants (Rensing *et al.*, 2008; Banks *et al.*, 2011; Brand *et al.*, 2013). However, members of Group IIa and III are exclusively found in angiosperms. Importantly, *AtWRKY40* and its close homologues *AtWRKY18/60*, identified as regulators of retrograde signalling in plants, are the only members belonging to the Group IIa WRKY gene family, which is specific to angiosperms (Supplementary Fig. S2) (Mohanta *et al.*, 2016). Therefore, Group IIa members are among the WRKY gene families which appeared most recently, evolved in angiosperms, and are suggested to originate from Group IIb genes (Liu *et al.*, 2012; Rinerson *et al.*, 2015; Cheng *et al.*, 2021). *AtWRKY63* belongs to Group III of the WRKY gene family, but we found that it is exclusively identified in the Brassicaceae family (Supplementary Fig. 2). In contrast, homologues/orthologues of *AtWRKY15*, also identified to regulate genes involved in MRR (Vanderauwera *et al.*, 2012), can be traced back to primitive vascular plants such as *Selaginella* and even non-vascular mosses such as *P. patens* (Supplementary Fig. S2)*. AtWRKY15* is thus the only member of mitochondrial signalling-related WRKYs found in *P. patens* and *Selaginella*, and is thus likely to be associated with mediating stress responses in early land plants. Other WRKY TF regulators of genes encoding chloroplast/mitochondrial proteins seemed to have appeared later in seed plants, or even relatively recently in Brassicaceae.

#### Cyclin-dependent kinase CDKE

**Fig. 2.**
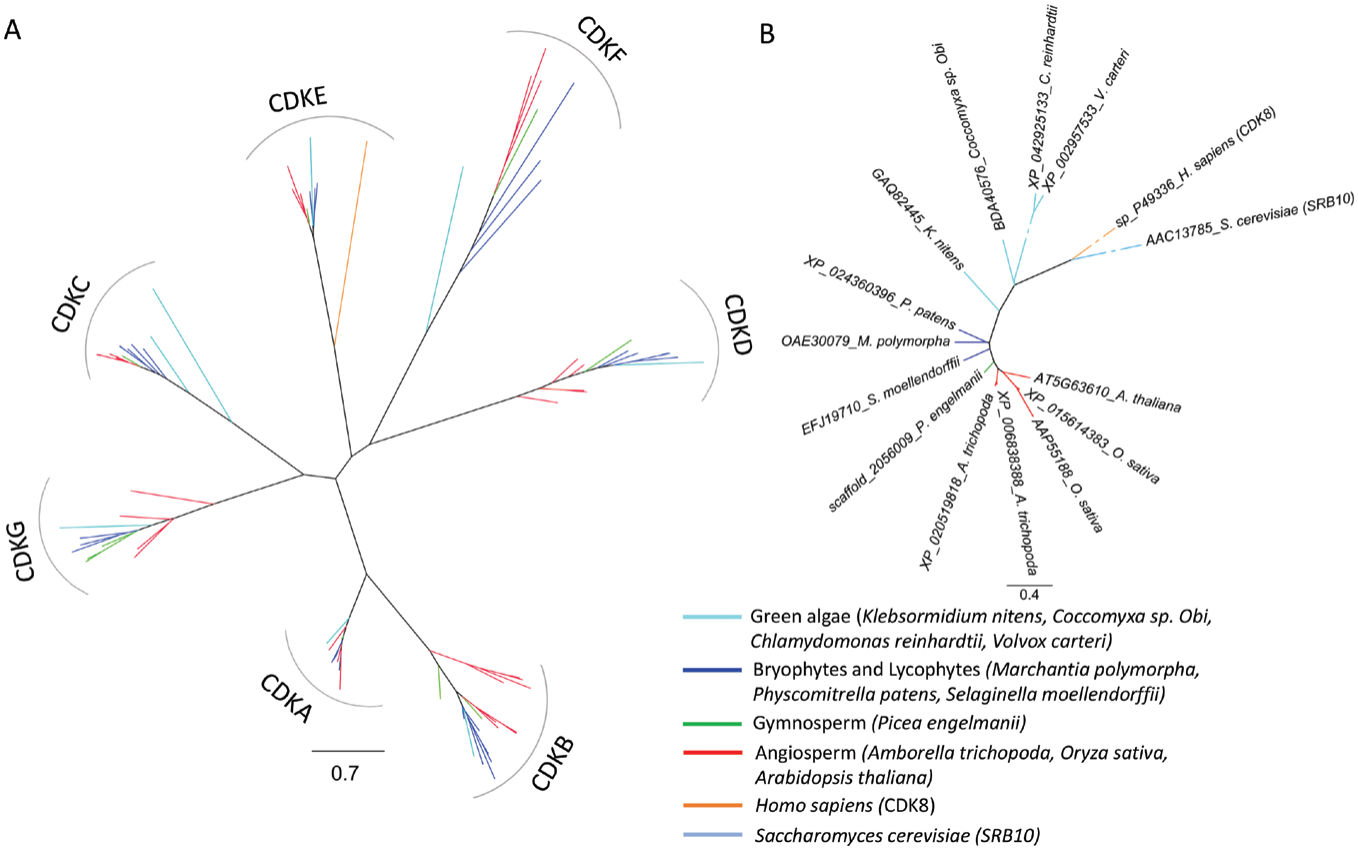
Phylogenetic relationship of CDK proteins. CDK protein sequences from representative streptophytes were identified by protein Blast searches using Arabidopsis as queries against the NCBI non-redundant protein database. Human CDK8 was obtained from the NCBI Gene Bank and also used in phylogenetic analysis. (A) Collected putative CDK proteins were aligned by MAFFT and the phylogenetic ML tree was constructed using IQ tree with 1000 bootstrap based on the full-length sequences (bootstrap values not shown for clarity). The unrooted tree representing CDK proteins from various species is grouped into CDK subgroups (CDKA to CDKG). (B) Phylogenetic analysis of CDKE proteins in similar plant groups, together with yeast Srb10 and human CDK8. Phylogenetic groups are indicated in different colour codes.

*CDKE;1* was the first gene reported as a positive regulator of *Alternative oxidase1a* (*rao1*) identified by a forward genetic screen using the Arabidopsis *AOX1a* promoter fused to a luciferase reporter gene (Ng *et al.*, 2013a). CDKs are highly conserved cell cycle regulators conserved in all eukaryotes. CDKE;1 (also known as CDK8) in Arabidopsis has been identified as a component of the Mediator complex, which is important for recruiting RNA polymerase II and regulating transcription (Zhu *et al.*, 2014). CDKE;1 can regulate gene expression dependent on or independently of its kinase domain. In the context of MRR, it was shown to be needed for induction of *AOX1a* gene expression in response to inhibition of Complex III by antimycin A (Ng *et al.*, 2013a). CDKE;1 also seems to be at the interface of chloroplast and mitochondrial retrograde signalling, by regulating the expression of light-harvesting complex B (*LHCB*) genes and *AOX1a* (Blanco *et al.*, 2014). Though CDK proteins are conserved throughout eukaryotes, the gene family make-up is relatively complex. For instance, the yeast *S. cerevisiae* only has six CDK homologues (though only CDK1/cdc28 is strictly required for mitosis) (Malumbres, 2014), while Arabidopsis has members in CDK subgroups from A to G. Furthermore, the kinase domains of CDKs are highly related to mitogen-activated protein kinases (MAPKs), with CDKs functionally defined by their interaction with cyclins. Using similarity searches, we tried to establish the origin of the CDKE subgroup. As shown in Fig. 2A, representatives of all CDK A to G subgroups can be found already in green algae, and were retained in land plants. We also included the human CDK8 and yeast srb10 proteins, which clearly shows that the CDKE subgroup is conserved from green algae to man. Though general CDKE domain conservation could be observed throughout eukaryotes, there are clear differences within the CDKE group. The analysed green lineage CDKEs (including green algae) have a completely conserved SPTAIRE motif (related to the PSTAIRE motif conserved in eukaryotic CDKAs), which cannot be found in, for example, human or yeast CDK8 (Supplementary Fig. S3). At the N-terminus, immediately before the kinase domain, only land plant CDKEs have a conserved WLQ(Q/H)Y motif, which is not found in green algae or more distant eukaryotes. Furthermore, we could only observe clear sequence similarity spanning 98–99% of the 471 amino acid Arabidopsis AtCDKE;1 protein sequence in the land plant lineage, including *P. patens* and *Selaginella*, but not in green algae or more distant eukaryotes. The closest *Chlamydomonas* homologue only covers ~70% of the AtCDKE;1 sequence, in the CDK domain, indicating that they are not true orthologues. The alignment between AtCDKE;1 and CDKE from the green alga *K. nitens* stretches from amino acids 18 to 367, and similar results were obtained for the green algae *Coccomyxa* sp. and *Volvox carteri*. The difference thus appears to be mainly in the C-terminus, with *K. nitens* CDKE having only 437 amino acids, as compared with AtCDKE;1 with 471. For the analysed land plant species, the length of CDKE was also tightly conserved from 467 to 471 amino acids. It thus appears that the CDKE subfamily has further evolved in conjunction with the colonization of land by Viridiplantae.

#### ANAC017-related NAC transcription factors

Without a doubt, the most prominent regulators of MRR in plants belong to the NAC transcription family, with ANAC017 the most important member in Arabidopsis. ANAC017 was identified as being involved in MRR using various screening approaches, together with related genes *ANAC013*, *ANAC016*, *ANAC053*, and *ANAC078* (De Clercq *et al.*, 2013; Ng *et al.*, 2013b). Later, studies confirmed ANAC017 as a key MRR master regulator in plants, with related genes such as *ANAC013*, *ANAC053*, and *ANAC016* making smaller contributions in positive feedback loops (Van Aken *et al.*, 2016a; Broda *et al.*, 2021). Interestingly, ANAC017 contains a C-terminal transmembrane motif (TMM) domain and is localized in the endoplasmic reticulum (ER), from where it translocates to the nucleus during mitochondrial perturbations caused by the environment or chemical inhibitors. There, it initiates mitochondrial retrograde responses by binding to the promoters of several mitochondrial dysfunction-responsive genes, including *AOX1a* (De Clercq *et al.*, 2013; Ng *et al.*, 2013b).

**Fig. 3.**
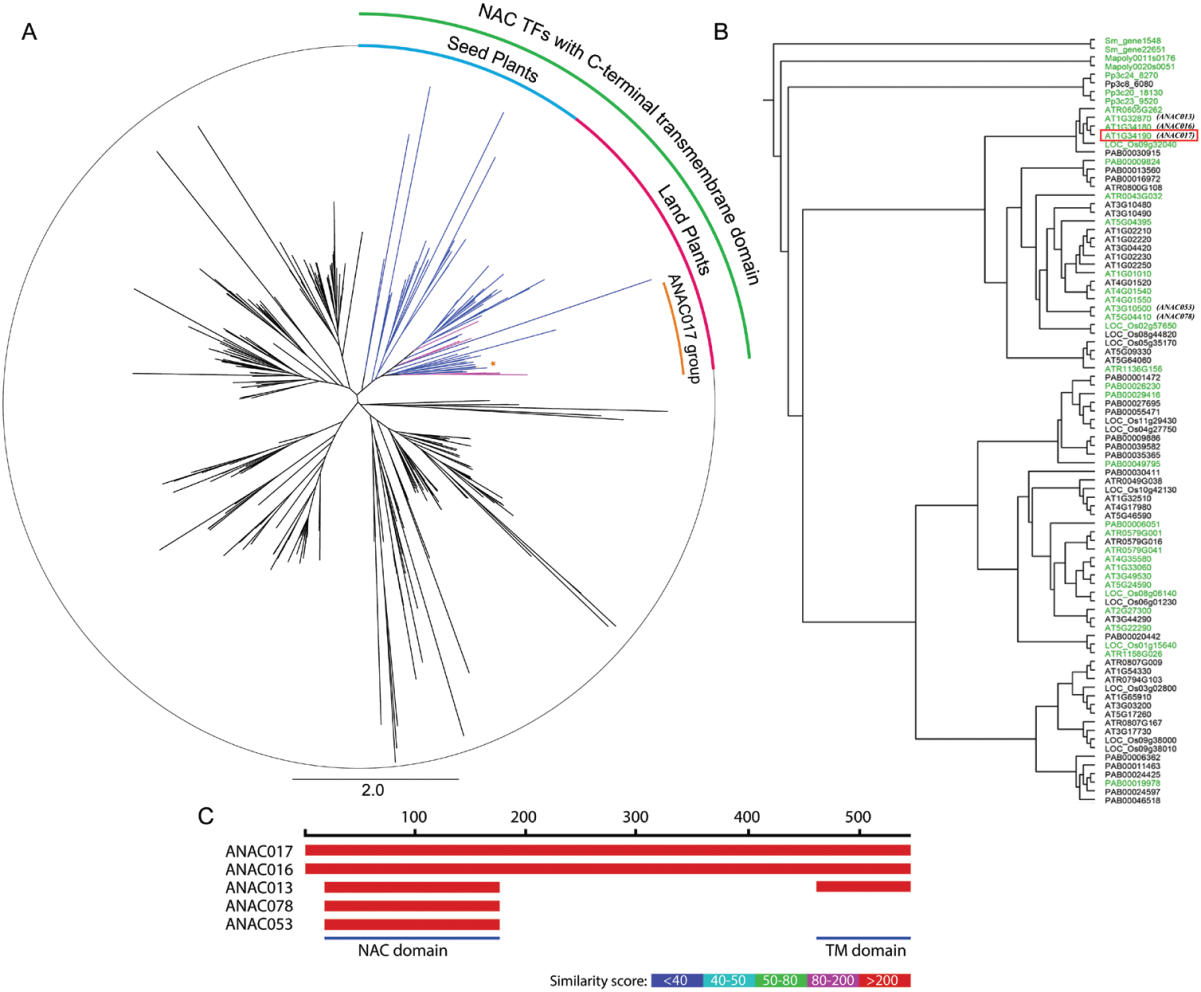
Phylogenetic analysis of the ANAC017-related transcription factors in land plants. (A) Unrooted phylogenetic tree of NAC genes from species representing the land plants including *Physcomitrium patens* (Pp..), *Marchantia polymorpha* (Mapoly..), *Selaginella moellendorfii* (Sm..), *Picea abies* (PAB..), *Amborella trichopoda* (ATR..)*, Oryza sativa* (LOC_Os..), and *Arabidopsis thaliana* (AT..). The clade of genes that contain C-terminal transmembrane motif (TMM) domains (or closely related genes that may have lost their TMM) are marked by the green circle section and with coloured branches. All land plant species contained at least two NAC TMM genes. The subgroup containing *A. thaliana* ANAC017 is indicated in orange and contains genes from all analysed seed plant species. ANAC017 itself is marked by the orange branch and asterisk. (B) Rooted phylogenetic tree focusing on the NAC domain-containing N-terminus of NAC TMM genes. Genes with predicted TMM domains are indicated in green. The five genes reported to be involved in MRR in Arabidopsis are named in parentheses, and ANAC017 is marked by the red box. Bootstrap values from 1000 runs were omitted for clarity. (C) Representation of the protein sequence alignment of ANAC017 and its closest Arabidopsis homologues, linked to MRR. The scale at the top indicates the amino acid position, and the colour scale represents the similarity score as found using the BLAST algorithm in www.arabidopsis.org, with red indicating the highest level of amino acid similarity, and lack of colour indicating low or no amino acid similarity. The approximate positions of the NAC and TMM domains are indicated. Note that ANAC078 and ANAC058 also have strongly predicted C-terminal TMM domains, but share very low sequence similarity with ANAC017 in this region.

Due to its role as a key factor involved in MRR in plants, we analysed the origin and evolutionary divergence of ANAC017 and its homologues in plants and green algae. The NACs form one of the largest plant-specific TF gene families, characterized by the presence of a conserved N-terminal NAC domain (DNA-binding domain) and a highly diverged C-terminal part, which together provide transcriptional regulation of target genes (Olsen *et al.*, 2005; Puranik *et al.*, 2012). Focusing on MRR regulators, here we assessed the evolutionary history of the NAC gene family in different green plant groups from ancestral algae to angiosperms. We found NAC gene sequences in all the assessed plant groups and in the green alga *K. nitens*. This is in line with the suggestion that the NAC family pre-dates the land plants (Maugarny-Cales *et al.*, 2016). Since the specific NAC TFs identified in mediating MRR responses are anchored into the ER by a C-terminal TMM, we focused our search on those NACs that possess C-terminal TMMs. Our phylogenetic analyses showed that C-terminal TMM-containing NAC TFs are clustered together in our unrooted tree (Fig. 3A) and can be found in all land plant groups from mosses to angiosperms. Absence of TMM-containing NACs in algae was further confirmed by searching for TMMs in individual NAC sequences. For instance, we found that the green alga *K. nitens*, thought to be related to the ancestor of land plants, does not yet contain C-terminal TMM NAC genes. Using Arabidopsis and rice NAC genes as reference, Pereira-Santana *et al.* (2015) also reported that Group III members from land plants exclusively represent the NAC genes that possess C-terminal TMMs. To further verify that the ANAC017-related TMM NACs appeared with land plants, we obtained the closest hit for ANAC017 in a wide range of plant species in a broad sense (Archaeplastida) present in the One KP database (One Thousand Plant Transcriptomes Initiative, 2019). In total, ANAC017-related protein sequences were recovered for 880 species. We created a phylogenetic tree of these 880 sequences, together with the 94 Arabidopsis NAC proteins (Supplementary Fig. S4). From this analysis, it was evident that indeed the TMM NACs appeared with land plants and cannot be found in green algae. Next, we narrowed down our search to known MRR regulators *ANAC017*, *ANAC013*, *ANAC016*, *ANAC053*, and *ANAC078*, and found that these NAC TFs are clustered together in one clade/subgroup together with genes from rice, the angiosperm sister group *Amborella trichopoda*, and the gymnosperm *Picea abies*, implying that the specific ANAC017-type TMM NACs have been present since at least early seed plants (Fig. 3B). As functional studies for TMM NACs are lacking in basal land plants, it is at present hard to estimate whether they were already involved in MRR from when they evolved, or whether this is a newer role acquired in seed plants. Other Arabidopsis members of this TMM-containing NAC group are also known to mediate stress signalling under a wide range of environmental stimuli, suggesting that these NACs may have played an important role in moderating MRR/stress responses during the transfer of aquatic plants to land (Kim *et al.*, 2007; Kim *et al.*, 2008; Seo and Park, 2010; Lee *et al.*, 2012).

As the NAC family is only well conserved in the NAC domain, we constructed a second tree focusing on the NAC domain-containing N-terminus of TMM NACs, while removing excess sequence gaps with more distantly related NAC members to obtain a more accurate local subtree (Fig. 3B). This phylogenetic tree clearly showed the increasing complexity of the TMM NAC family in vascular plants, with 2–4 TMM NAC genes in mosses and *Selaginella*, while seed plants contained much higher numbers (e.g. 30 in Arabidopsis and 22 in *P. abies*). A closer inspection of the presence of a predicted TMM using TM-HMM (Krogh *et al.*, 2001) showed that many of the annotated genes were truncated and/or had lost the TMM domain, while others retained a clear TMM signature (genes marked in green in Fig. 1B). ANAC017 itself was found in a small subgroup with its duplicated gene ANAC016 (Broda *et al.*, 2021) and ANAC013 from Arabidopsis, and one representative of rice (Os09g32040), *Amborella*, and *P. abies*. Interestingly, the annotated *P. abies* gene in the ANAC017 subclade seemed to have lost its TMM domain. This may be due to incorrect gene annotation or an effective loss of the domain. ANAC078 and ANAC053 were located further away in a much larger subclade containing many genes for all analysed seed plants. As it was previously shown that ANAC017 and ANAC013, but also ANAC053 and ANAC078, could bind the *AOX1a* promoter (De Clercq *et al.*, 2013) and upon overexpression trigger a higher constitutive expression of *AOX1a*, *UPOX*, and other MRR target genes (Van Aken *et al.*, 2016a), it is thus likely that this entire group has potential factors involved in MRR. Interestingly, when comparing the alignments of ANAC017 with its closest homologues in Arabidopsis, it became clear that ANAC017 and ANAC016 are nearly identical, but ANAC013 is only highly conserved in the N-terminal NAC domain and the C-terminal TMM domain (Fig. 1C). Even more strikingly, the sequence similarity between ANAC017 and ANAC078/053 was only high around the NAC domain (Fig. 1C). Although ANAC078/053 still have strongly predicted TMM domains at the corresponding position, their actual sequence was quite divergent.

In Arabidopsis, ANAC017 is by far the most highly expressed gene of these related NACs, while the others are more weakly expressed and in fact under *ANAC017* expression control to form a positive feedback loop (Van Aken *et al.*, 2016a; Broda *et al.*, 2021). Therefore, it would be relevant to measure the expression patterns of *ANAC017/78*-related genes in other species to identify the most dominant regulators. Notably, the TMM and membrane-anchoring mechanism probably provide a rapid and controllable transcriptional response system under fluctuating environmental conditions, and hence may have played a significant role in mediating stress responses during evolution of land plants (Hoppe *et al.*, 2001). Phylostratic co-expression analysis also indicated that the ANAC017-dependent MRR regulon dates back to as early as land plants (Lama *et al.*, 2019). In conclusion, it is clear that the TMM NAC family evolved together with the colonization of land, and strong evidence exists that TMM NACs involved in MRR date back to at least the arrival of seed plants. However, functional characterization of the TMM NACs from mosses and early vascular plants is required to demonstrate their specific role in MRR control and their significance in terrestrial plant colonization. The ANAC017-related MRR pathway is also still evolving, as exemplified by the relatively recent incorporation of mitochondrially targeted *DUF295* genes into the ANAC017 pathway in Brassicaceae (Lama *et al.*, 2019).

## Discussion

### MRR: an essential tool developed for the conquest of land?

**Fig. 4.**
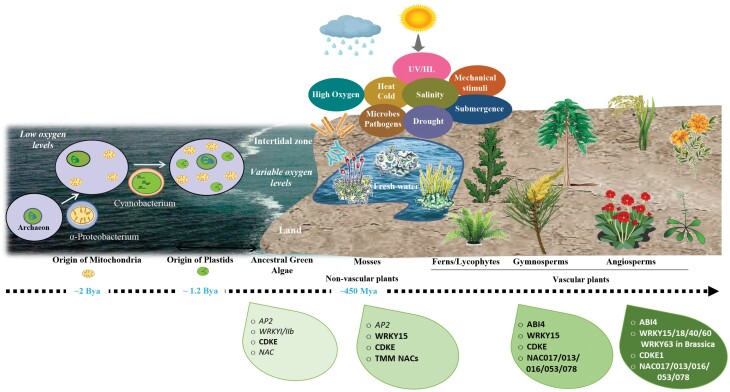
The appearance of mitochondrial retrograde regulators during the evolution of land plants. Eukaryotes are thought to have evolved by endosymbiosis between a host archaeon and an aerobic prokaryotic cell (related to α-proteobacteria), which gradually resulted in a mitochondrion (Dyall *et al.*, 2004; Embley and Martin, 2006; Khachane *et al.*, 2007; Martin *et al.*, 2015). In photosynthetic eukaryotes, this was followed by acquisition of free-living photosynthetic cyanobacteria, which led to evolution of specialized plastids (Nisbet and Sleep, 2001; Martin and Russell, 2003; Dyall *et al.*, 2004; Pereira-Santana *et al.*, 2015). These events of endosymbiosis gave rise to the group of glaucophytes, rhodophytes, and chlorophytes. The chlorophyte subgroup of charophytes (photosynthetic freshwater green algae) probably contained the ancestor that diversified into the land plants. Major environmental pressures including drought, salinity, desiccation, UV irradiation, drastic temperature shifts, variable oxygen levels, mechanical stimuli, and submergence were associated with their movement from the aquatic environment to land. These ancestral streptophytes possessed various biochemical/physiological/genetic traits such as basic gene families of present-day regulators of retrograde signalling (green boxes shown at the bottom). During the course of evolution, these emerging early plants evolved traits adaptive to land, resulting in non-vascular plants (mosses), and later on tracheophytes and seed plants. This explosion of land plants further increased the oxygen levels to current levels. One part of the adaptations was probably the improved communication and interaction between mitochondria and nuclear gene expression via retrograde signalling, with more complex regulatory networks arising as vascular and eventually flowering plants developed. Green boxes at the bottom represent the evolution of gene families (AP2/ABI4, WRKY; CDKs/CDKE1: cyclin-dependent kinases, and NAC gene families), containing known regulators of mitochondrial retrograde signalling in modern plants. In all cases, the basic gene families were present in ancestral green algae, but large expansions occurred together with the transition to land, including formation of C-terminal transmembrane NAC genes (TMM NACs), formation of the CDKE land plant group, and WRKY15-type proteins. With seed plants, came the development of the ANAC017-type proteins and ABI4-like proteins. WRKY40/18/60-type genes appeared relatively late in the angiosperms, and WRKY63-type genes are even more recent, apparently having evolved in Brassicaceae. These observations suggest that the transition to land was a major driver for development of mitochondrial signalling in plants, and that further fine-tuning of the regulatory network developed during the more recent evolution of land plants and is likely to be still ongoing.

The above phylogenetic analysis of factors involved in MRR revealed a clear pattern, with many of the major players apparently having evolved in the common ancestors of present-day land plants. This thus suggests that a range of factors associated with transition to land were drivers to evolve and/or diversify mitochondrial retrograde signalling in plants. The evolutionary history of photosynthetic eukaryotes showed a wide range of algae that successfully established themselves on land (Souffreau *et al.*, 2013; Edwards, 2014; Nishiyama *et al.*, 2018). The first land plants no doubt found the terrestrial environment to be a bountiful source of carbon dioxide and light resources that may have limited growth and reproduction of their aquatic algal ancestors (Fig. 4) (Graham, 1993). At the same time, there was probably a stressful trade-off in the form of increased temperature fluctuations, desiccation, drought, oxidative damage, UV-B, salinity, xenobiotics, and hypoxia. To meet these challenges while maintaining cellular homeostasis, early land plant relatives must have acquired sensor and signalling networks to overcome changing climatic conditions. In this section, we will discuss the probable role of retrograde signalling in combatting these environmental challenges and how these triggers have diverged during land colonization.

### MRR to mitigate changes in oxygen and CO_2_ availability

Since early land plants probably grew in shallow water on the boundaries of ponds or along the banks of meandering rivers, they were greatly influenced by variations in oxygen supply caused by cyclic floods and drought. Moreover, when plants initially invaded terrestrial habitats, atmospheric oxygen levels were greater than in the original aquatic environment, and the explosion of land plant growth further promoted the rise of oxygen, which reached present-day levels by 400 MYA (Scott and Glasspool, 2006; Alboresi *et al.*, 2010; Lyons *et al.*, 2014; Lenton *et al.*, 2016; Dahl and Arens, 2020). Increased atmospheric oxygen levels with a greater exchange rate than in water, along with limited oxygen availability during floods/submergence, significantly influenced metabolic activities in early land plants. Certainly, oxygen deprivation directly inhibits mitochondrial function as it is a key substrate for the mitochondrial electron transport chain and alters the dynamics of reactive oxygen species (ROS), NAD^+^/NAD(P)H and ATP/ADP, membrane potential, and calcium. Oxygen deprivation has thus been suggested to lead to MRR induction (Schwarzlander and Finkemeier, 2013; Wagner *et al.*, 2019; Van Aken, 2021). In agreement, inhibition of Complex III under normal conditions by antimycin A shows a large overlap in transcriptomic pattern between target genes of MRR and hypoxia, suggesting that NAC-dependent signalling plays a significant role in the response to low oxygen stress (Wagner *et al.*, 2019). Recent studies have now experimentally proved that ANAC017-mediated mitochondrial retrograde signalling enhances plant survival under hypoxia, flooding, and submergence (Bui *et al.*, 2020; Meng *et al.*, 2020). Also, AtWRKY40 and AtWRKY45, both of which can bind the *AOX1a* promoter, were found to be positive regulators of flooding tolerance (Meng *et al.*, 2020). Overexpression of mitochondrial uncoupling protein (UCP) in Arabidopsis also triggered a hypoxia-like response mediated by the cytosolic N-degron pathway, further suggesting the MRR pathways to have a close link to hypoxia survival (Barreto *et al.*, 2022). Most of the studies suggested mitochondrial ROS as the main retrograde signal in mediating mitochondrial responses during hypoxia, but the complete understanding of retrograde signal(s) is still a work in progress (Chang *et al.*, 2012; Ng *et al.*, 2013b). However, in animal systems, ROS signals originating from the mitochondrial electron transport chain are confirmed to elicit adaptive responses in response to hypoxia (Hamanaka *et al.*, 2016).

Organisms progressively moving from, for example, coastal to more inland biotopes may thus have been facing periods of submergence followed by periods of air exposure, for instance in intertidal zones or after heavy rainfall/flooding. Such an environment may require a much higher flexibility when it comes to mitochondrial respiration than in stable aquatic or stable air-exposed environments. Perhaps then it is no surprise that AOXs are one of the best understood targets of MRR in plants and have been used extensively to identify the upstream factors involved in MRR (Schwarzlander *et al.*, 2012; Ng *et al.*, 2013b; Van Aken *et al.*, 2016b). AOX operates as a second terminal oxidase in the electron transport chain of plant mitochondria, bypassing Complex III and IV and transferring the electrons directly to oxygen from ubiquinone. As a result, it maintains the redox homeostasis by minimizing ROS generation during mitochondrial perturbations/MRR and hence enhances plant performance under adverse conditions (Maxwell *et al.*, 1999; Millenaar and Lambers, 2003; Selinski *et al.*, 2018). However, preventing the overproduction of ROS via AOX respiration comes at the cost of lower energy production capacity due to absence of proton transport across the membrane. A number of studies have demonstrated the importance of AOX in plant adaptions against a range of abiotic and biotic stressors, reviewed in the following sections (Saha *et al.*, 2016; Vanlerberghe *et al.*, 2016; Dahal and Vanlerberghe, 2017) and described further below.

The presence of AOX is sometimes thought to be restricted to plants, but it is widely distributed in fungi, protists, and the animal kingdom as well. The presence of *AOX* genes in ancestral α-proteobacteria suggests the endosymbiotic origin of AOX in eukaryotic lineages (Finnegan *et al.*, 2003; McDonald, 2008). Furthermore, the ancestral origin of AOX in eukaryotes probably played a vital role in the evolution of advanced multicellular organisms during the transition to land. The identification of AOX sequences in ~150 taxa representing the majority of the animal kingdom’s invertebrates (McDonald and Gospodaryov, 2019) further demonstrates its evolutionary significance in adaptation and selection during the development of terrestrial ecosystems. A recent study on a marine copepod (*Tigriopus californicus*), which lives in shallow splash pools, showed an induction in *AOX* at the mRNA and protein level at various developmental stages of the animal as well as in response to cold and heat stress (Tward *et al.*, 2019), suggesting that *AOX* may also be an MRR target gene in animals. It seems that AOX has been lost in animals relying on relatively fast-moving lifestyles to hunt and avoid being caught, such as present-day vertebrates (McDonald and Gospodaryov, 2019). Most probably, the non-energy-conserving AOX pathway was too energetically wasteful to be kept for these animals relying on higher mobility. As a trade-off, vertebrates seem to have lost oxygen metabolism flexibility and thus require more consistent oxygen availability. For non-mobile plants, linking AOX into the MRR network was thus likely to have been an important step during land transition to coordinate such metabolic flexibility.

Another major target of the plant MRR pathways are alternative NADH dehydrogenases (NDs), which provide a non-proton pumping alternative to Complex I (Rasmusson *et al.*, 2008; Van Aken *et al.*, 2016b). Also here, NDs are much more widespread than in plants, with homologues in fungi, but also in some primitive animals, for example Cnidaria and Bilateria such as Echinodermata. Overall, these findings indicate the importance of AOX/ND during early eukaryotic evolution and adaptation in various stages of oxygenated life on earth. As land plants strongly rely on oxygen metabolism flexibility, for instance during germination in relatively hypoxic soil (Merendino *et al.*, 2020; Jurdak *et al.*, 2021), but also later on during flooding events, it is clear that an efficient regulation by MRR that balances energy loss with survival has been an important requirement for successful colonization of land by plants. Prevention or removal of ROS is another important function of the MRR pathways in plants, to which AOX and NDs contribute (Maxwell *et al.*, 1999; Smith *et al.*, 2011). H_2_O_2_-responsive gene 1 (HRG1; *At2g41730*) is another ANAC017 target gene and was recently shown to reduce oxidative stress, further underlining the role of MRR in regulating oxygen usage and toxicity in land plants (Gong *et al.*, 2021).

Furthermore, since the emergence of photosynthetic eukaryotic algae from freshwater and potentially from lake margins, the biosphere has been progressively oxygenated, with a general decreasing trend in relative CO_2_ levels. These events led to evolution of photorespiration in green lineages and played a significant role in colonization of land plants (Cummins, 2021). As mitochondria are essential parts of plant metabolism and photorespiration, and are thus closely intertwined with photosynthesis, it is plausible that these environmental alterations might have been major triggers to evolve retrograde signalling pathways in early land plants (Becker and Marin, 2009; de Vries *et al.*, 2016).

### MRR in prevention of water loss and hyperosmotic stress

In terrestrial habitats, a steady water supply is no longer a given. Early land plants thus developed wax layers and other adaptations such as roots, vasculature, and stomata to prevent excessive water loss. Also, temperatures and UV/VIS radiation input are much more variable on land than in the ocean, affecting water availability and photosynthesis. Mitochondria and plastids have been suggested to act as key components in sensing and responding to drought, salinity, heat, and high light stress conditions. To date, several studies have established that drought and high light stress are primary inducers of chloroplast retrograde signalling, which results in the activation of acclimatory cellular responses (Chan *et al.*, 2016; de Vries *et al.*, 2016; de Vries and Archibald, 2018). One such response involves 3ʹ-phosphoadenosine 5ʹ-phosphate (PAP) as a retrograde signalling molecule to initiate ABA-mediated responses and stomatal closure under severe stress condition (Pornsiriwong *et al.*, 2017). Increased ROS generation under oxidative stress leads to inactivation of SAL1 (inositol polyphosphate 1-phosphatase) due to the formation of disulfide bonds, and therefore inhibits degradation of PAP into AMP (Estavillo *et al.*, 2011). Accumulated PAP in chloroplasts moves to the nucleus and activates the transcriptional response. Localization of SAL1 protein and accumulation of PAP also in mitochondria provides evidence for metabolic interaction and overlapping functions of mitochondria and chloroplasts during stress conditions (Estavillo *et al.*, 2011). Furthermore, the SAL1–PAP- and ANAC017-mediated retrograde signalling pathways regulate the expression of overlapping target genes, suggesting that these two signalling pathways are likely to be convergent (Van Aken and Pogson, 2017). Homologues of SAL1–PAP signalling components have been found in ancestral streptophyte algae, suggesting that early land plants most probably utilized SAL1–PAP as an operational retrograde signal to land-specific stresses (Zhao *et al.*, 2019). In the same study, the early evolutionary role of SAL1–PAP signalling during transition to land was demonstrated in ABA-mediated stomatal regulation in land plants including mosses, ferns, and flowering plants.

In agreement with a role for mitochondrial retrograde signalling, ANAC017 and many of its target genes have been associated with drought and salinity resistance, including *AOX1a*, *UDP-glycosyltransferase UGT74E2*, and *Outer mitochondrial membrane protein of 66 kDa* (*AtOM66*) (Giraud *et al.*, 2008; Skirycz *et al.*, 2010; Tognetti *et al.*, 2010) (Zhang *et al.*, 2014). MRR transcriptional regulators can also directly impact on drought tolerance, as *atwrky63* and *anac017* mutants were found to be more sensitive to drought (Ren *et al.*, 2010; Ng *et al.*, 2013b). It is thus likely that development of MRR contributed to facilitating the transition to land of early plants with regard to water availability and osmotic stress.

### MRR: booster of the ancient immune system?

Microbes were associated with the precursors of land plants, green algae, and aided in the transition of algae from aquatic to terrestrial environments, indicating that microbes were critical in the evolution of plants (Lyu *et al.*, 2021). These interactions could have been beneficial for the plants, with microbes helping plants to explore nutrients from the environment more efficiently (e.g. mycorrhiza and rhizobia). On the other hand, there has been a continuous competition and arms race between plants and pathogenic microbes during terrestrialization. Studies in early land plants showed major plant defence mechanisms built on phenylpropanoid-mediated biochemical defences that are conserved in land plant gene families of distant lineages (Berland *et al.*, 2019; Jiao *et al.*, 2020). Genomic and transcriptomic studies in ancestral chlorophytes suggested the common ancestral origin of the nucleotide-binding site-leucine-rich repeat (NBS-LRR) defence system in green plants. Furthermore, major defence phytohormones including jasmonic acid, salicylic acid (SA), and ethylene have been detected in members of streptophyte algae (Hori *et al.*, 2014; Ju *et al.*, 2015; Beilby *et al.*, 2015; Van de Poel *et al.*, 2016). This suggests that the key components needed for plant–microbe interactions were already in place, ready to expand and evolve to assist with land colonization.

Mitochondrial involvement in pathogen infection and host responses has been suggested to potentially amplify the first ‘alarm signal’ in order to trigger the defence responses at the cellular level (Colombatti *et al.*, 2014). Elevated ROS levels during pathogen perception act as signalling molecules and initiate defence responses. Pathogen-associated molecular pattern compounds such as harpin are known to disrupt mitochondrial function and induce overproduction of mitochondrial ROS (Krause and Durner, 2004). It is thus not unexpected that mitochondrial function and ROS production, which are at least in part co-regulated by MRR, are associated with microbial interactions in plants. Some NBS-LLR receptors also have mitochondrial and plastidial transit peptides (Goyal *et al.*, 2020; Wang *et al.*, 2020). Plant mitochondria are known targets of plant pathogens, with some fungi even producing effectors that directly target mitochondria to help them in their infection by, for instance, suppressing excessive ROS production (Han *et al.*, 2021).

Over the past years, many of the regulators and target genes of MRR in plants have been associated with pathogen defences. This further suggests that MRR was an integral part of the capacity of plants to survive on land. As described above, mitochondrial dysfunction induces the alternative pathway by up-regulating *AOX1a* and *NDB3/4* gene expression via the ANAC017 MRR pathway (Van Aken *et al.*, 2016b), and similar responses were observed during pathogen infection (Cvetkovska and Vanlerberghe, 2012b; Vanlerberghe, 2013; Huang *et al.*, 2016). The role of *AOX1a* has already been established in plant defence against bacterial, viral, and fungal pathogens via maintaining ROS levels (Cvetkovska and Vanlerberghe, 2012a). Additionally, SA, which is primarily involved in the defence of plants against biotic and abiotic stressors, induces the expression of *AOX1a* via an unknown mechanism, which may involve MRR (Xie and Chen, 1999; Norman *et al.*, 2004; Gleason *et al.*, 2011; Belt *et al.*, 2017; Zhang *et al.*, 2020). SA may operate at the ubiquinone-binding site of succinate dehydrogenase and enhance SA-induced transcriptional responses by ROS production at Complex II (Belt *et al.*, 2017). Notably exogenous SA also induces MRR and alternative pathway genes (Ho *et al.*, 2008). Overexpression of *AtOM66* triggered high basal SA levels, SA-induced responses, and accelerated programmed cell death rates, and provided resistance against necrotrophic pathogens. *AtOM66* appears to enhance SA production by up-regulating SA biosynthetic genes in overexpressing plants (Zhang *et al.*, 2014). It thus seems that MRR may have contributed to let plants resist the ongoing pressure by microbial pathogens. For instance, it has been suggested that limiting excessive mitochondrial ROS production during pathogen infection is important for survival and to prevent autoimmunity (Huang *et al.*, 2013). Interestingly, it has been found that mitochondria can move inside the cell to the sites where pathogens are trying to invade, and may assist in defence by producing localized ROS that fight off the pathogen (Fuchs *et al.*, 2016). Thus, it seems that balancing mitochondrial ROS production at the right time and place is important for surviving pathogen attack, and thus may be partially controlled by MRR networks. A tight co-evolution of increased diversity of plant–microbial interactions with MRR is thus likely to have been of importance during terrestrialization.

### Concluding remarks

Because the majority of studies examining the role of MRR are focused on angiosperms, our understanding of MRR is limited from an evolutionary perspective. The elucidation of these components and their associated signalling pathways in various groups of plants, such as present-day gymnosperms, mosses, algae, and others, would provide detailed information on the evolution of these networks across lineages. Nonetheless, based on the available genomic and transcriptomic data, we observed that the broader gene families of the present-day factors involved in MRR in Arabidopsis, such as WRKY, NAC, ABI4, and CDKE factors, were already present in the ancestral green algae (Fig. 4). This indicates that basic physiological and genetic components for developing current retrograde signalling in streptophytes were available and may have facilitated their transition to land under adverse climatic conditions. However, when looking in more detail, it appears that the transition to land required a massive expansion of these gene families and signalling networks to deal with the new environment. In agreement, key factors involved in MRR such as the ANAC017 group probably only evolved from ancestral NACs (found in at least some green algae) during this dramatic period of moving to land-based habitats (Fig. 3). Also, in the case of ABI4 and the ANAC017-type regulators, we observed that specific subtypes associated with MRR may have appeared in seed plants (Fig. 1 and 3). As experimental evidence for factors involved in MRR in bryophytes is lacking, it is difficult to assess if these seed plant-specific modifications were needed to facilitate their role in specifically MRR, and if bryophytes may have developed their own complex MRR systems. Perhaps the evolutionary development of dehydrated seed structures, which are often associated with disassembly of mitochondria during the maturation of the seed, created an additional pressure to develop or expand MRR networks. Seeds often germinate in the soil under hypoxic conditions, which may also have been a driver to further develop MRR in seed plants. Furthermore, MRR target genes and regulators have also been associated with a range of other physiological processes that have evolved in land plants, including senescence (Meng *et al.*, 2019; Broda *et al.*, 2021), and responses to mechanical stimuli (Van Aken *et al.*, 2016a; Xu *et al.*, 2019) and UV (Pascual *et al.*, 2021).

As mitochondrial retrograde pathways found in other distant kingdoms such as fungi and animals appear to use genetically unrelated signalling components compared with plants (Tran and Van Aken, 2020), one wonders how the earliest eukaryotes that contained mitochondria controlled retrograde signalling. This raises the question of whether those ‘paleo-MRR’ pathways were lost or are just so poorly understood or generalized that we have not been able to pinpoint them in present-day organisms. At the very least, it is probable that the new environmental cues caused by transition to land were a primary trigger for developing and expanding retrograde signalling in early terrestrial plants. Additional fine-tuning events appear to have taken place in seed plants (spermatophytes) or even more recently. Those needs and pressures, as well as available lineage-specific TF families that had already been established, would have been very different for animals, for example, so it can be easily imagined that their transition to land led evolution of MRR in a very different direction. Taken together, this work provides an evolutionary perspective on mitochondria to nucleus signalling and portrays how retrograde signalling is intertwined with green plant terrestrialization and evolution of photosynthetic eukaryotes.

## Supplementary data

The following supplementary data are available at *JXB* online.

Fig. S1. Sequence alignment of ABI4 homologues.

Fig. S2. Phylogenetic tree of WRKY transcription factors.

Fig. S3. Sequence alignment of CDKE homologues.

Fig. S4. Phylogenetic analysis of ANAC017/transmembrane domain-containing NACs in Archaeplastida.

Dataset S1. FASTA files, alignments, and phylogenetic tree information for CDKE, ABI4, and NAC transcription factors.

erac351_suppl_supplementary_figures_S1-S4

erac351_suppl_supplementary_dataset_S1

## Data Availability

The datasets used in this study are available in the Supplementary data.
