# Supplementary material for: The colonization of land was a likely driving force for the evolution of mitochondrial retrograde signalling in plants
Source: J Exp Bot. 2022 Sep 3;73(21):7182–97. doi: 10.1093/jxb/erac351 (PMC9675596; doi:10.1093/jxb/erac351)
Supplement: erac351_suppl_supplementary_figures_S1-S4 [file erac351_suppl_supplementary_figures_s1-s4.pdf]

Supplementary data

The colonization of land was a likely driving force for the evolution of mitochondrial retrograde signalling in plants

Kasim Khan and Olivier Van Aken*

Department of Biology, Lund University, Lund, Sweden

*Corresponding author: Olivier Van Aken
Molecular Cell Biology
Department of Biology
Lund University
Sölvegatan 35
Lund 223 62 – Lund, Sweden
Tel: +46 76 210 14 03
e-mail: olivier.van_aken@biol.lu.se

ORCID: Olivier Van Aken (0000-0003-4024-968X); Kasim Khan (0000-0001-7336-2764)

Highlights

Phylogenetic analysis shows that the transition to land was a major driving force for developing mitochondrial retrograde pathways in plants, while additional fine-tunings appeared in seed plants or even later.

Keywords

Mitochondria, chloroplasts, retrograde signalling, plants, colonization of land, evolution, stress response

```
K. nitens #1       M-------------------------------------------------LPTPSDKP-------------------------------------------
K. nitens #2       MDADLFLLLDSSAQDSFEFLNATKELIPKGQAISALEESALSGPARSSISVEVQGQEPERLTRRAQDHSQAANLEASVSLLCHEGNTEILYSNPVTISRRE
C. reinhardtii     M-----MHLDAKAKHSLLALDAASLGL------------ALPGTSPPAAPLPVPGVKP-------------------------------------------
M. polymorpha      M----------------------------------------------------------------------------------------------------
P. patens          M----------------------------------------------------------------------------------------------------
S. moellendorffii  M----------------------------------------------------------------------------------------------------
P. taeda           M----------------------------------------------------------------------------------------------------
T. plicata         M----------------------------------------------------------------------------------------------------
A. trichopoda      M----------------------------------------------------------------------------------------------------
A. thaliana        M----------------------------------------------------------------------------------------------------
O. sativa          M----------------------------------------------------------------------------------------------------

K. nitens #1       -----------------------------------GSQTD-------------------------------------------------------------
K. nitens #2       EESIDESSGEGMEAAQGGSPASQEMKQNSTAEGEPGACTILQAETINESRTENLTTTLETTGTPEDGQAVRASARGEHLTPLSEAFRTVDTGRPNGLLCVA
C. reinhardtii     ------PGAAGVAALPPGSLPF-------------GAATQF-------------------------GQ-----------VPF---FAGLPFPLPGG-----
M. polymorpha      -----------------------------------------------------------------------------------------------------
P. patens          -----------------------------------------------------------------------------------------------------
S. moellendorffii  -----------------------------------------------------------------------------------------------------
P. taeda           -----------------------------------------------------------------------------------------------------
T. plicata         -----------------------------------------------------------------------------------------------------
A. trichopoda      -----------------------------------------------------------------------------------------------------
A. thaliana        -----------------------------------------------------------------------------------------------------
O. sativa          -----------------------------------------------------------------------------------------------------

K. nitens #1       ---------------ETHDAATVAS-------------IIDAYYGPHARSED--------ARTLNFLNDQGP----------------------------
K. nitens #2       AKRTQLPTLLDNSPPQQPASEDGQAPKRLGFAEKKFGRDFESFIGEERATGTLGENPDLRAGSLDQAEDRGPVERSLGGVRGFKKGLRTAFLSRDRARDA
C. reinhardtii     -----VPPPLPGLPPLSSAAAAAAA-------------------AAHAHAAAAAHA----AAAMTIAQAAAAAEAAPGKGR-------------------
M. polymorpha      -----------------------------------------------------------------QLNDT------------------------------
P. patens          ------------------------------------------------------------------------TEIAMG----------------------
S. moellendorffii  ----------------------------------------------------------------------------------------------------
P. taeda           ----------------------------------------------------------------------------------------------------
T. plicata         ----------------------------------------------------------------------------------------------------
A. trichopoda      ----------------------------------------------------------------------------------------------------
A. thaliana        ----------------------------------------------------------------------------------------------------
O. sativa          ----------------------------------------------------------------------------------------------------

K. nitens #1       DG--------------------------RRPNTDASHPGSSG------------PASTPPTAGSSDLTQQGS---FLHREKGPISPAV-----AVRSSPG
K. nitens #2       DGFFSARRLPRAARAGDPVSTQAGVQSDQRSEALPGQPLSNGLKKFDGVFPEGSPAEAPGPFDRELLTSLGIGKDSLERAAGSSPTAVLS-PRALVAQPP
C. reinhardtii     IGAIAA----SAAAATDP------------NIAVPTPPIAAS------------PPTEPSD-GSSPATSHGS-------KDGAAGDAVKTEPGANTAAPG
M. polymorpha      ----------------------------------------------------------------------------------------------------
P. patens          ----------------------------------------------------------------------------------------------------
S. moellendorffii  ----------------------------------------------------------------------------------------------------
P. taeda           ----------------------------------------------------------------------------------------------------
T. plicata         ----------------------------------------------------------------------------------------------------
A. trichopoda      ----------------------------------------------------------------------------------------------------
A. thaliana        ----------------------------------------------------------------------------------------------------
O. sativa          ----------------------------------------------------------------------------------------------------

K. nitens #1       EE-EGITKKNESLKAAVGGIHSPLARGS----AAFEGGPGSGEVAGEVREQAALRSLLAEFQGS--PADLLALMQRDEEAEEAEIEEGGSGLRRVTSSGS
K. nitens #2       DS-LSLAHLSPPLQAYQGNTNFPNHYHSPSNVPAFAHSPSALDFLNS--------MQLPPFEPSRAPPSLSQFLQRPEVT---------------RSEDS
C. reinhardtii     GALSSLGAIAAAAQAATAGMAVPP--------SLWLGQPGSAGSAFQ-----ALAMPMPPF-----PPNLA-----------------------RTSVGG
M. polymorpha      ----------------------------------------------------------------------------------------------------
P. patens          ----------------------------------------------------------------------------------------------------
S. moellendorffii  ----------------------------------------------------------------------------------------------------
P. taeda           ----------------------------------------------------------------------------------------------------
T. plicata         ----------------------------------------------------------------------------------------------------
A. trichopoda      ----------------------------------------------------------------------------------------------------
A. thaliana        ----------------------------------------------------------------------------------------------------
O. sativa          ----------------------------------------------------------------------------------------------------

K. nitens #1       GGAARMFDVEPLFSGGMMEALLGRMEEQRADEDVSSPAKKMILRNSSLQVGKVLSPGS-EGQSRISHDDPMKTLSTTPFPLRRSEEKPPNEKGGIEQQLV
K. nitens #2       GGELDAAMEAPPIS-------MAVRQSMESDLAPPLPAEPQVTKEAA------------PPTSAPHQQDPYNR---------------PDL---------
C. reinhardtii     FGAEPAEEAAPGISG--FKSMLRKKQQVRPGKSTEPNALPVILEGKPVELNRRVSASPKSPMDLVESDDPVGGAEDV-----NGRRKQPGR-G------V
M. polymorpha      DGSVQPGTSAP--------A---------HSDAPSAPA----------------PSGR-PPLA-------------------------PGMPG-------
P. patens          --------------------------------------------REELSSDRVD-----DPQCEDAHGEGSGG--------------DKARAG------A
S. moellendorffii  --------------------MARQQQQQQHSASATSSCVPVFDKQEEDQTPLV------VPEQLEAHHDHVDS---------------------------
P. taeda           ------------------------------DNSAPPPT----------------PPSP-PPPA-------------------------------------
T. plicata         ----------------------------------------------------------------------------------------------------
A. trichopoda      ----------------------------------------------------------------------------------------------------
A. thaliana        -----------------------------------------------------------ASLSHQNQN--------------------------------
O. sativa          -----------------------------------------------------------DPLA-------------------------------------

K. nitens #1       TEEPAQRDPVVADTISLSGGTKRRRLSERKRP---ENSTSR----ENGKQLYLGGFDSEEQAALAYDIAAIKCRGASAQTNLALENYRAHFDDISAVSKD
K. nitens #2       KRPAANSPSAVGKRSSQYRGVTKHCSTGRYEAHLWDNSSRKEGINKKGRQVYLGGYEKETDAARAYDLAALKYWGVGTNYNFPLKDYEQDLKEMEDVSRQ
C. reinhardtii     -----NLPSSLADHPAAGICSTSYRSSGSHLG-------------MPGGAL------GASPPSLGTSPAAIKLPGRRRTSSTNL---------LSSSLGA
M. polymorpha      TTATASQQQQQQQQLQLVAAAASGGGGGGAAA-------------AAGAR--------------------------------------------------
P. patens          ----NSDTNSSASDVDSKTGVATADTKTRGRK-------------KKGTAA------------------------------------------SSSSGSG
S. moellendorffii  ----PIQPPGVAEEVSSAAAGAAATSNARPSS-------------QKGGR--------------------------------------------------
P. taeda           --------KGGNREAQCSAVGQESGGHSSPTT-------------APPST--------------------------------------------------
T. plicata         ----------VGEESGVRTGQEHTTAEAQPEA-------------KSG----------------------------------------------------
A. trichopoda      ----QNQQHENPSPSSSPSSSHSSKSSERNSS-------------GEG----------------------------------------------------
A. thaliana        ----SQHQHNHLEDNNQTLTHNNPQSDSTTDS-------------STSSA--------------------------------------------------
O. sativa          ------EPSDDACTVAAPAAETAASSSGAGGG-------------GGGGRT-------------------------------------------------
```

AP2-associated motif

KGGP(D/E)NXKFR

AP2-Domain

```
K. nitens #1        ELVMSLRRHSKGFS-RGSSKFRGV-TRHQKGRWEARIGQLQGKKYMYLGLYDTEEEAAAAYDREAIRQKGHHAVTNFDISL LLSQQASQAVQRQQEQQRA
K. nitens #2        EFVATLRRKSSGFS-RGASKYRGVTRHHQHGRWEARIGRVLGNKYLYLGTYPTQEEAAQAYDIAAVKYRGVHAVTNFDLSR YLEFMRPGG---------S
C. reinhardtii      KASSSVPSLSVKPDKSGACKYRGV-RQRPWGKYAAEIRDPHKGCRLWLGTYDTAEEALAYDKAAREIRGPRAVVNFPNVN H-----------------A
M. polymorpha       ---RGRGKGKGGPQ-NGLFQYRGV-RQRSWGRWVAEIREPRRRARIWLGTFATAVDAAHAYDCAAWRLYGPRARLNLA--- -------------------
P. patens           NGQERVRKRRGGPE-NGLHQYRGV-RQRQWGRWVAEIREPRLRTRMWLGTFSTALEAARAYDEAALVYHGPGARLNLP--- -------------------
S. moellendorffii   ---RARARGKGGPE-NGLYTYRGV-RQRSWGRWVAEIREPRRRTRVWLGTFATAQDAAQAYDMAAWRLYGSRAQLNLR--- -------------------
P. taeda            ---KRKCKRKGGPD-NIKFQYRGV-RQRSWGKWVAEIRQPGKRTRRWLGTFATAEQAAQAYDNAAILLYGSKAHLNLQ--- -------------------
T. plicata          ---RRKCKGKGGPD-NNKYRFRGV-RQRSWGKWVAEIREPRKRTRKWLGTFSSAEEAARAYDRAAALMYGPRAQFNLQ--- -------------------
A. trichopoda       ---RRKCKGKGGPD-NNKFRYRGV-RQRSWGKWVAEIREPRKRTRKWLGTFSTAEEAARAYDRAAAALYGARAQLNLE--- -------------------
A. thaliana         ---QRKRKGKGGPD-NSKFRYRGV-RQRSWGKWVAEIREPRKRTRKWLGTFATAEDAARAYDRAAVYLYGSRAQLNLT--- -------------------
O. sativa           ---KKKAAGKGGPE-NGKFRYRGV-RQRSWGKWVAEIREPRKRSRKWLGTFATAEDAARAYDRAALLLYGPRAHLNLT--- -------------------
```

Serine/Threonine-rich region

```
K. nitens #1        QSLQPPPFAPPPHAQLPPTSPP-------------------------------------SLQASPRYAPPRARTPPSRPHSS---PRQVRSPSLR--SP-
K. nitens #2        QTLPAPPSADSEGGTGAEEGSD-------------------------------------LTYGSPRKRKRAGSERPAARSFE---PRVLSTQIV---MP-
C. reinhardtii      NLPAPQPHGDDESGAWDPLGTT-------------------------------------SLGTSPVSSGFIPGSSPLMGG-----SAPVRHGMH----PH
M. polymorpha       YPISSQPSDSPPLESCIQTS---------------------------------------QAS---TSNSSSCSLKPLLPAIA-PRPNLAHVQSQ------
P. patens           ------HETTQKSENVPQLGNQGVEYHYGHLKTNKFMGLGVPANLLIGSDVASTDKYFMSVDTSRLRQSSSCSVENI------GQPAGFNLNRD----PE
S. moellendorffii   ------PSPPSFSSSSRQGS---------------------------------------------AGGSSVAKPRIIL-------PSSMRPRMMS--LP-
P. taeda            ------PSGWDQS----------------------------------------------------KSSSHSSKLRPLLPRITVTRPPAIHGTIP------
T. plicata          -----PPIHPP------------------------------------------------------ASARSSSTLRPLLPR-----PPNMAPRGG------
A. trichopoda       -----QPVASKLT----------------------------------------------------APQSSAASLRPLLPR-----PTGFNTGFNH--HI-
A. thaliana         -----PSSPSSVSSSSSSVS---------------------------------------AASSPSTSSSSTQTLRPLLPR-----PAAATVGGG------
O. sativa           ---APPPLPPPPPSSAAAAA---------------------------------------ASSSSAASSTSAPPLRPLLPR-----PPHLHPAFHH--QP-
```

LRP motif

```
K. nitens #1        --GASNRLRPQASSPTFS-----------------------------LSAALGQTPP-------------------------------------GSPPYH
K. nitens #2        --SPAPRF-PEPASPTEP-----------------------------ASPY--QVDP-------------------------------------RIDPRY
C. reinhardtii      --HHLHGGAFSHHPGVAPSSGLGLRSTFGGYPV--------------------RREP-------------------------------------------
M. polymorpha       ---------CQPPPAAAP-------------------------------------MP--HHLLDFRHVEALVPAAIPMPAAS-----------LRDAAGW
P. patens           WWISCGSFRPNSIPQSVPNFSNVPPSTSSCNVGSERFHLSQVPINFQTIPISCKLPPDRHELVQGFPVPQQLSAGLFHSKYTQTLGLRSP----NIHCEL
S. moellendorffii   --PHHHFQIPNFNQ-----FLSHQAATSSLF-----------------------PLP---NATAATAVAASGAAKVSGRLV-------------------
P. taeda            ------------GPNPNPNISGAFPCGYFGT----------------------LTIP---DFWPAAMRAAHTDITYHHP---------------VIDPKR
T. plicata          ---------------PSRFVYAGFPQFNS------------------------SIIP--PEVSVAVPVGV-APQYVLYPPLTDQIPHGSPDLSRSQVPKV
A. trichopoda       ----------NAYPCSASTFSL-------------------------------LNNP-----TSSLGSCNQFPATVSQ-------------FPVPVSPYV
A. thaliana         ---------ANFGP---------------------------------------YGIPFNNNIFLNGGTSMLCPSYGFFPQQQQQ---------QNQMVQM
O. sativa           --FHHHLLQPQPPPPPPPLYYAATASTSTVTTTT-------------------TAPP--PQLAAAAPAAVLVAAAVSSTAETQAVVATAP----EDAASA

K. nitens #1        RSQSWPRTQPLS------------GPYVGLGGFAPVGPQ-------------------------------------------------------------
K. nitens #2        APLSVDDQQALS------------DQFYFGGEFSPTLDQ-------------------------------------------------------------
C. reinhardtii      ------------------------TEPIAEGESDVDMDD-----------------GEEGED------------GDGDDMDEGVGPRSGRARMKVNVVTG
M. polymorpha       HLYSRSSEKSLIGENFADD--KS-KVRLLLGAPAPLNSK-GRTQFQSPNLYDCCRGVPDCCSSIARDENFRIHGRESEIDVPGLSESTAVVGLAAQLMT-
P. patens           GSDCPYADRQGRGLHRNDT--HNTGAVMFAKSSTTQTDS-FFESNQNEPL--------------------KVPIGDASP---------------------
S. moellendorffii   ----------------------------------------------------------------------------------------------------
P. taeda            ETTLEQRSVFHLEESRVPTQQCN-GTEVLHRESSPLKTDKLQENFRTENT---------------RLQSHRL--GDPDQLSGIQGGIVGDE-LSQGFYS-
T. plicata          KSNQSTKDATLVEEIAAAKRGKD-DREI---SQQPALDL---CTSQNGLL---QQNGHVCNG------------GDYEVYSNGMSRCTGDQNLPESFIP-
A. trichopoda       DSTQQPLIHGFYDEIGGLA--GSVDASL---SLSRVSDE---SLSHPRVSGAETQGYEVRNG------------GEREI---------------------
A. thaliana         GQFQHQQYQNLHSNTNNNK--IS-DIEL---TDVPVTNS---TSFHHEVALGQEQGGSGCNN------------NSSMEDLNSLAGSVG------SSLS-
O. sativa           AAAAAAEEEAAWGFHGGDE--EDYAAALLWSEPDP-----------------------------------------------------------------

K. nitens #1        ------PSAA----------------------------VPADVSLEQAALWRSVFG------------QPGGAHVGWGNVP-------------------
K. nitens #2        ------PFSPLNTTHAVHLPNPLQYCQLV---------PYPNLSLNFPQALS----------------QLFPVYPGLGQ---------------------
C. reinhardtii      VGGRTRPSRPAAAAAVAAAAAAAAGGG-VDLEDSDGAPPPPQPSMA------------------------------------------------------
M. polymorpha       ANSTHGLSDACSLIERVEAAMQ--------IQDLHHE-ALRSQKLHLDAV-----------QSRERELQRNSSAPGSLEFRPIKAEPQERAATDYSHLQQ
P. patens           DMAPTFTATPHQPEQPVAVLVPGES------TAMFNPPPPSDVSCQ------------------------------------------------------
S. moellendorffii   ----------------------------------------------------------------------------------------------------
P. taeda            -----ETAGNITEGESRDDNLVASL------HELQYSGGPPSPGFM------------------------------------------------------
T. plicata          LMHCPNPAAPKDVLEQVSVSIPPEY------GEFKHS-VPPSPALM------------------------------------------------------
A. trichopoda       --------------PAVSMAVP----------------PSPSSSLM------------------------------------------------------
A. thaliana         ITHPPPLVDP-----VCSMGLDPGYM------------VGDGSSTI------------------------------------------------------
O. sativa           ----------------------------------------------------------------------------------------------------

K. nitens #1        ------------------MPQPS----------------------------------------------------------------PSEFQPGAMPPQ-
K. nitens #2        ------------------YPQTL----------------------------------------------------------------PGAYAPGLQVGS-
C. reinhardtii      ----------------------------------------------------------------------------------------------------
M. polymorpha       APLDAVVPPLVCSSDQFRWPDTVQPTAASREADASIA--ACKEWWHPI---AR-QILNSCSPE--------------------FES-ADEYTVPSTATAV
P. patens           ------------------YCSNV-------------E--APKKFECPI---SEGSAENSFVTE--------------------FEKFDFESDAFQSMDSS
S. moellendorffii   ----------------------------------------------------------------------------------------------------
P. taeda            ------------------WHYNIKH--------------------------------------------------------------DYYYDETKSST--
T. plicata          ------------------W----------------------------------------------------------------VGGSDYYYDNGMVSSS-
A. trichopoda       ------------------WP-------------------------------------------------------------------PYEYEQAAMLSA-
A. thaliana         ------------------WP---------------------------------------------------------------FGG-EEEYS--------
O. sativa           ----------------------------------------------------------------------------------------------------

K. nitens #1        --QRPAKLVA-----------RRGPVEA-------PPLAAGSPPLGSGG---GTGGAQ------------PVPQTEV-ESTRPGLAARRRMLRRRKMALS
K. nitens #2        -------------------DNREGAEDA-------VHASNGLVP--------------------------------------------------------
C. reinhardtii      -----------------------------------------------GGASRGGGGK----------------------RNQVSMDVEDEL---------
M. polymorpha       QAQQHPSLLWN--------SPEHSLSSS-------PSVTS------------------------------PMPPTMIWNSSEQSLTSPPSVTS---S---
P. patens           IESQHDKLVSGVTAA--TAVMDHG-----------PPVSSEASPVSLQGADFGDEGSPS----------SFWPDSTT--SSHQSLNASESC------PWS
S. moellendorffii   ----------------------------------------------------------------------------------------------------
P. taeda            -----------------------------------------------------------------------LPETD------NQL---------------
T. plicata          ---------------------------------------------------------------------HSMGNME---SDNGPL---------------
A. trichopoda       -----------------------------------------------------------------------LPPMD---PNPSSL---------------
A. thaliana         -------------------------------------------------------------------------------HNWGSI---------------
O. sativa           ----------------------------------------------------------------------------------------------------
```

```
K. nitens #1       QMYHQQRQIDDLELVAPAEQS----LQSQPSPYPGQEQPLEQEHLQGLGGLHPSS---------YE-------------------------
K. nitens #2       ----------------PGYSA----WDAAQ-------------------------------------------------------------
C. reinhardtii     -------------------------AELADALLL----------------LHESA------------------------------------
M. polymorpha      --------------SHPILAD--GQWEYLPASGSMSNNTLN--------CCHSDACNCSCSFMDYDSFMHIMQ*-----------------
P. patens          NIQEERYMLK----LCPVFEDMNGCWDDAGSPILPPPSLLDLPDLPN---------------LSFDSLTEVFCDTPKVNDVDKAANFPDL*
S. moellendorffii  -------------------------------------------------------------------------------------------
P. taeda           -------------------------WDYSDESSI---------------------------------------------------------
T. plicata         -------------------------WDN---DFI------------------------------FDP*-----------------------
A. trichopoda      -------------------------WEDGE-PFM------------------------------FDL------------------------
A. thaliana        -------------------------WDFID-PIL----------------------------GEFY*------------------------
O. sativa          -------------------------W--------------------------------------FDLFLK---------------------
```

Supplementary Figure 1: Multiple sequence alignment of obtained ABI4 proteins from the representative members of different evolutionary green plant group. ABI4-specific conserved domains, AP2-associated motif (yellow), the AP2 domain (grey), the serine/threonine-rich region (pink) and the LRP motif (cyan) were identified and indicated in mentioned colour schemes.

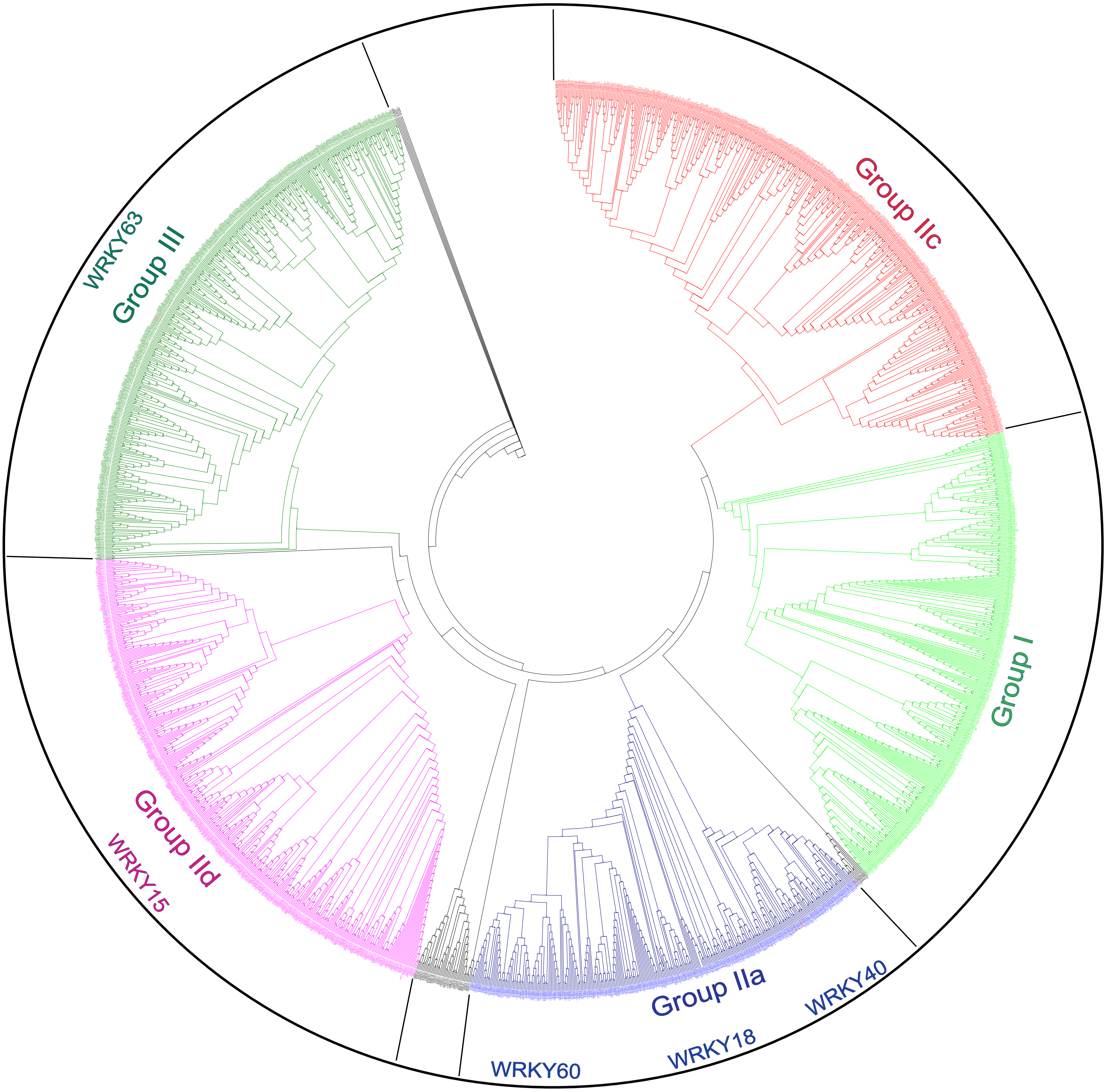

***Supplementary Figure 2:* Phylogenetic analysis of WRKY transcription factors.**

The unrooted phylogenetic tree showing evolution of WRKY TF family/groups in lower eukaryotes, monocots and dicots was obtained from Mohanto et al., 2016. We used this tree to show the evolution of major WRKY TFs including WRKY15, (Group IId) WRKY18, 40, 60 (Group IIa) and WRKY63 (Group III) involved in the regulation of mitochondrial retrograde responses in plants.

```
A. thaliana            ----------------------------------------------------------MGDGSSS---RSNSSNSTSEKPE
O. sativa#1            ----------------------------------------------------------MGDGRVG---------GGTNRPA
A. trichopoda#1        ----------------------------------------------------------MGDGT------------RSNRPA
A. trichopoda#2        ----------------------------------------------------------MGDGT------------RSNRPA
O. sativa#2            ----------------------------------------------------------MGDGRVG---------GGTNRPA
P. engelmanii          -----------------------------------------------------------NNNN------------NSNRPA
S. moellendorffii      ----------------------------------------------------------MADGIA-----------KRARPA
M. polymorpha          M-----RSLRSAV---------------------GAWLAFERLLLRVPVP-----SWNLGPGNPR----------QRARPA
P. patens              M---------------------------------------------------------VANGSVK----------QRTRPE
K. nitens              --------MDR-----------------------------------------------SGGGTAS----SAARAKAVARPS
Coccomyxa sp. Obi      --------MSTHN-------RSKYAPPQKDN---------------AP---------------------STVQDPLAAVPA
C. reinhardtii         --------MNIQL--------------QRGQQY----DS------QAQR---------GGQGQGGTQNQQAQQNQTAATPP
Volvox carteri         MPVCHPHTINLRLVNTIFVNPPGRIPPQRNNPP----NPFCHLPFQIPRLVHPP---LAG--------------RASEQPP
H. sapiens (CDK8)      --------MDYDF--------------------------------------------------------KVKLSSERERVE
S. cerevisiae (SRB10)  M---YQRPMQVQGQ-------------QQAQSFVGKKNTIGSVHGKAPMLMANNDVFTIGPYRAR---------KDRMRVS

A. thaliana            WL------QQYNLVGKIGEGTYGLVFLARTKTP---------PKR--P---------------------------------
O. sativa#1            WL------QQYELVGKIGEGTYGLVFLARLKQS-HPHAAAGVGRRGSP---------------------------------
A. trichopoda#1        WL------QQYDLVGKIGEGTYGLVFLAKSKLA---------SNKGKC---------------------------------
A. trichopoda#2        WL------QQYDLVGKIGEGTYGLVFLAKSKLA---------SNKGKC---------------------------------
O. sativa#2            WL------QQYELVGKIGEGTYGLVFLARLKQS-HPHAAAGVGRRGSP---------------------------------
P. engelmanii          WL------QHYDLVGKIGEGTYGLVFLARSKLP---------NNRGLR---------------------------------
S. moellendorffii      WL------QQYELLGKIGEGTYGLVYLAKSKLV---------SNRGVK---------------------------------
M. polymorpha          WL------QQYELVGKIGEGTYGLVYLARRQQP---------LHRGVR---------------------------------
P. patens              WL------QQYELVGKIGEGTYGLVYLARSKEP---------AHRGTK---------------------------------
K. nitens              WW------DLYEVVGKIGEGTYGLVYLVRVKDK----------AQGYK---------------------------------
Coccomyxa sp. Obi      YLS-----EHYTVIDKIGEGTYGVVYMAKTKDQ-----------HPRM---------------------------------
C. reinhardtii         ISSSSSVWDKFEKGDKIGEGTYGLVYHARSKET------------GGR---------------------------------
Volvox carteri         ISPAGSPTPLLP-----GEGTYGLVYHARSKET------------GGR---------------------------------
H. sapiens (CDK8)      DL------FEYEGC-KVGRGTYGHVYKAKRKDG----------KDDKD---------------------------------
S. cerevisiae (SRB10)  VL------EKYEVIGYIAAGTYGKVYKAKRQINSGTNSANGSSLNGTNAKIPQFDSTQPKSSSSMDMQANTNALRRNLLKD

A. thaliana            ----------------------------------IAIKKFKQSKDG------DGVSPTAIREIMLLREISHENVVKLVNVH
O. sativa#1            ----------------------------------IAIKKFKQSKEG------DGVSPTAIREIMLLREINHENVVKLVNVH
A. trichopoda#1        ----------------------------------IAIKKFKGSKDG------DGVSPTAIREIMLLREISHENVVKLVNVH
A. trichopoda#2        ----------------------------------IAIKKFKGSKDG------DGVSPTAIREIMLLREISHENVVKLVNVH
O. sativa#2            ----------------------------------IAIKKFKQSKEG------DGVSPTAIREIMLLREINHENVVKLVNVH
P. engelmanii          ----------------------------------IAIKKFKQSKDG------DGVSPTAIREIMLLREFSHENVVKLVNVH
S. moellendorffii      ----------------------------------IAIKKFKQSKEG------DGVSPTAIREIMLLRECMHENVVKLVDVH
M. polymorpha          ----------------------------------IAIKKFKQSKEG------DGVSPTAIREIMLLRECIHDNIVKLVNVH
P. patens              ----------------------------------IAIKKFKQSKEG------DGVSPTAIREIMLLRECVHENIVKLVNVH
K. nitens              ----------------------------------LAIKKFKPSKDG------DGVSPTAIREIMLLRECCHENIVKLVNVH
Coccomyxa sp. Obi      ----------------------------------LAIKTFKPGKEG------DGISPTAIREIMLLRELKHDNIVHLDAVH
C. reinhardtii         ----------------------------------YAIKQFKGGREG------DGVSPTAIREIMLLREMRHPNIVKLESAY
Volvox carteri         ----------------------------------YAIKQFKSGREG------DGVSPTAIREIMLLREMSHPNIVKLESAH
H. sapiens (CDK8)      ----------------------------------YALKQI----EG------TGISMSACREIALLRELKHPNVISLQKVF
S. cerevisiae (SRB10)  EGVTPGRIRTTREDVSPHYNSQKQTLIKKPLTVFYAIKKFKTEKDGVEQLHYTGISQSACREMALCRELHNKHLTTLVEIF

A. thaliana            INFADMSLYLAFDYAEYDLYEIIRHHR----DKVG-HS------LNTYTVKSLLWQLLNGLNYLHSNWIIHRDLKPSNILV
O. sativa#1            INHADMSLYLAFDYAEHDLYEIIRHHR----EKLN-LP------INPYTVKSLLWQLLNGLNYLHSNWIIHRDLKPSNILV
A. trichopoda#1        INHSDMSLYLAFDYAEHDIYEIIKHHR----EKVS-QP------IKEYTVKSLLWQLLNGLNYLHSNWIIHRDLKPSNILV
A. trichopoda#2        INHSDMSLYLAFDYAEHDIYEIIKHHR----EKVS-QP------IKEYTVKSLLWQLLNGLNYLHSNWIIHRDLKPSNILV
O. sativa#2            INHADMSLYLAFDYAEHDLYEIIRHHR----EKLN-LP------INPYTVKSLLWQLLNGLNYLHSNWIIHRDLKPSNILV
P. engelmanii          INHVDMSLYLAFDYAEHDLYEIIRHHR----EKLNHHS------INQYTVKSLLWQLLNGLNYLHSNWIVHRDLKPSNILV
S. moellendorffii      INHADMSLYLAFEYAEHDLYEIIRHHR----EKLL-FQ------INPYTVKSLLWQILNGINYLHSNWIIHRDLKPSNILV
M. polymorpha          INYVDMSLYLAFDYAEHDLYEIIRHHR----EKLS-SP------INQYTVKSLLWQILNGLNYLHSNWIIHRDLKPSNILV
P. patens              INHGDMSLYLAFDYAEHDLYEIIRHHR----EKVT-CS------INPYTVKSLLWQILNGLNYLHSNWIIHRDLKPSNILV
K. nitens              INHADASLYLAFDYAEYDLYEIIRFHR----EKLQ-YA------ISPYTVKSLMWQILNGLHYLHSNWIIHRDLKPSNILV
Coccomyxa sp. Obi      LHRPEPSLSLAFDYAEHDLYEMLRFHR----ERGM-GP-----VLETYTLKSLMWQLLDGLQYLHSNWVMHRDLKPSNILV
C. reinhardtii         INRSEPSLWLAFEYAEYDLYEMIKFHR----DHKENTRDNPFGLMPQYIVKTVMWHLLNGLSYMHQHWVVHRDLKPSNVLV
Volvox carteri         INHSEPSLWLAFEYAEYDLYEMIKFHR----DNKDNRRDNPFGLMPQYIVKTVMWHLLNGLSYMHQHWVVHRDLKPSNVLV
H. sapiens (CDK8)      LSHADRKVWLLFDYAEHDLWHIIKFHRASKANKKP-VQ------LPRGMVKSLLYQILDGIHYLHANWVLHRDLKPANILV
S. cerevisiae (SRB10)  LER--KCVHMVYEYAEHDLLQIIHFHS----HPEK-RM------IPPRMVRSIMWQLLDGVSYLHQNWVLHRDLKPANIMV

A. thaliana            M-GDA-----EEHGIVKIADFGLARIYQAPLKPL-SD-NGVVVTIWYRAPELLLGSKHYTSAVDMWAVGCIFAELLTLKPL
O. sativa#1            M-GEG-----EEHGIIKIADFGLARIYQAPLKPL-SD-NGVVVTIWYRAPELLLGAKHYTSAVDMWAVGCIFAELLTLKPL
A. trichopoda#1        M-GEG-----DEQGVVKIADFGLARIYQAPLKPL-SD-NGVVVTIWYRAPELLLGAKHYTSAVDMWAVGCIFAELLTLKPL
A. trichopoda#2        M-GEG-----DEQGVVKIADFGLARIYQAPLKPL-SD-NGVVVTIWYRAPELLLGAKHYTSAVDMWAVGCIFAELLTLKPL
O. sativa#2            M-GEG-----EEHGIIKIADFGLARIYQAPLKPL-SD-NGVVVTIWYRAPELLLGAKHYTSAVDMWAVGCIFAELLTLKPL
P. engelmanii          M-GEG-----EEHGVVKIADFGLARIYQAPLKPL-SD-NGVVVTIWYRAPELLLGAKHYTSAVDMWAVGCIFAELITLKPL
S. moellendorffii      MSGDG-----DEQGLVKIGDFGLARIYQAPLKPL-SD-NGVVVTIWYRAPELLLGAKHYTCAVDMWAVGCIFAELLTLKPL
M. polymorpha          M-GDG-----EEQGVVKIGDFGLARVHQAPLKPL-CD-NGVVVTIWYRAPELLLGAKHYTSAVDMWAVGCIFAELLTLKPL
P. patens              M-GEG-----EEQGVVKIGDFGLARIFQAPSRPL-SD-NGVVVTIWYRAPELLLGSKHYTSAVDMWAVGCIFAELLTLKPL
K. nitens              M-REG-----DEAGVVKLGDFGLARIYQAPLKPL-SD-NGVVVTVWYRSPDLLLGAKHYTPAVDVWAVGCIFAELLTLKPL
Coccomyxa sp. Obi      V-GEG-----EEQGRVKIGDFGLARIYACPLRPL-SD-NGVVVTIWYRAPELLLGARHYTPAADVWAAGCILAEMLALRPL
C. reinhardtii         M-GEDPAVAPHQHGCVKIADFGLARIFQSPARPL-SD-NGVVVTIWYRAPELLLGARHYTRAVDVWAAGCIFAELLTLKPL
Volvox carteri         M-GEDPAVAPSQHGCVKIADFGLARIFQAPARPL-SD-NGVVVTIWYRAPELLLGARHYTRAVDVWAAGCIFAELLTLKPL
H. sapiens (CDK8)      M-GEG-----PERGRVKIADMGFARLFNSPLKPL-ADLDPVVVTFWYRAPELLLGARHYTKAIDIWAIGCIFAELLTSEPI
S. cerevisiae (SRB10)  T-ID---------GCVKIGDLGLARKFHNMLQTLYTG-DKVVVTIWYRAPELLLGARHYTPAVDLWSVGCIFAELIGLQPI
```

```
A. thaliana            FQ-GAEAKSSQN---PFQLDQLDKIFKILGHPTMDKWPTLVNLPHWQNDVQHIQAHK--YDSVGLHNVVHLNQK-------
O. sativa#1            FQ-GVEAKATPN---PFQLDQLDKIFKVLGHPTVEKWPTLANLPCWQNDQQHIQGHK--YENTGLHNIVHLPQK-------
A. trichopoda#1        FQ-GVEVKATPN---PFQLDQLEKIFKVLGHPTQEKWPTLVNLPCWQNDQQHIQSRK--YDNPGLHSVVHLPQK-------
A. trichopoda#2        FQ-GVEVKATPN---PFQLDQLEKIFKVLGHPTQEKWPTLVNLPCWQNDQQHIQSRK--YDNPGLHSVVHLPQK-------
O. sativa#2            FQ-GVEAKATPN---PFQLDQLDKIFKVLGHPTVEKWPTLANLPCWQNDQQHIQGHK--YENTGLHNIVHLPQK-------
P. engelmanii          FQ-GVEVKASPN---PFQLDQLDKIFKVLGHPTPEKWPTLMNLPHWTRNLQQIQQRK--YDNAGLH-IGPIPPK-------
S. moellendorffii      FQ-GAEDKTGPN---PFQLDQLDKIFKVLGHPTTERWPMLSNLPHWLANRQLIQSRK--YDNPGLHTVVNLQPK-------
M. polymorpha          FQ-GIEDKNGQN---PFQLDQLDKIFKTLGHPTADRWPTLLNLPFWQANRNLIQGRK--YENPGLYSIVQQPAK-------
P. patens              FQ-GIEDKSSPS---PFQFDQLDKIFKVLGNPTADKWPTLTTLPHWAQNRQSIQSRK--YDNPGFYSIVQLPPK-------
K. nitens              FQ-GVEDKGMHN---PFQIDQIDKIFKVLGPPSVDTWPGLAHLPYWQHNRQSIQNKK--YDHPLLPSMTAHFAK-------
Coccomyxa sp. Obi      FQ-GNERKTPAS---AFQADQCDKIFRVLGLPSAASWPALEYLPHWRDNTENVRAHK--PEFPAKSRLAEVIAEYSALAAG
C. reinhardtii         FQ-GQERKTPGN---VFQADQLDKIFRVLGHPSIKNWPELEVLPHWVENTDNVRVKRPEWSGTGLHTAVLEAMR-------
Volvox carteri         FQ-GQERKTPGN---VLQADQLDKIFRVLGHPGIKTWPELEVLPHWVDNTDNVRVRRPEWGGTGLHSAILEAMR-------
H. sapiens (CDK8)      FHCRQEDIKTSN---PYHHDQLDRIFNVMGFPADKDWEDIKKMPEHSTLMKDFRRNT--YTNCSLIKYMEKHKV-------
S. cerevisiae (SRB10)  FK-GEEAKLDSKKTVPFQVNQLQRILEVLGTPDQKIWPYLEKYPEYDQITKFPKYRD--NLATWYHSAGG-----------

A. thaliana            -------------------SPAYDLLSKMLEYDPLKRITASQALEHEYFRMDPLPGRNAFVASQPMEKNVNYPTRPVDTNT
O. sativa#1            -------------------SPAFDLLSKMLEYDPRKRITAAQALEHEYFRMDPLPGRNALLPSQAGEKIVQYPVRPVDTTT
A. trichopoda#1        -------------------TPAYDLLSKMLEYDPRKRVTAAQALEHEYFRIEPPPGRNALVPSQPGEKAVNYPARPVDTTT
A. trichopoda#2        -------------------TPAYDLLSKMLEYDPRKRVTAAQALEHEYFRIEPPPGRNALVPSQPGEKAVNYPARPVDTTT
O. sativa#2            -------------------SPAFDLLSKMLEYDPRKRITAAQALEHEYFRMDPLPGRKLERKLCNILCVQLIPQLILKEQQ
P. engelmanii          -------------------SPAYDLLSKMLEYDPRKRITAAQALDHEYFRIDPQPGRNALVPSQPGEKAINYPPRSVDANT
S. moellendorffii      -------------------GLAFDLLSRMLEYDPVKRITAAQALDHEYFRSDPLPGRNALVYGQPGEKVVQYPARPVDSST
M. polymorpha          -------------------SPAFDLLSKMLEYDPAKRISAAQALDHQYFRTEPLPGRNALVPGSPGEKVVHYPARPVDYST
P. patens              -------------------SPGFDLLSKMLEYDPIKRITAAQALEHEYFRNDPIPGRNSLVPANSADKVVPYPQRPVDYST
K. nitens              -------------------TAAYDLLTKMFEYDPAKRLTAEQALKHEYFRQEPLPGKNSFVSGYPGEKVVVYPSRPVDTSL
Coccomyxa sp. Obi      GGANPS---------DQRGGALSDLLSSMLAYNPEHRISAADALQHPYFQEEPRPGRNAFIHN--GRLLVQYPRRSKHGPA
C. reinhardtii         -GAAPSWAQGHVPQECVPPREAIDLMHRMLDFNPSTRTTAEECLRHEWFRQEPKPGPNVFRLPGASTPLVRYPRRPMSLDR
Volvox carteri         ---------------------AIDLMSRMLDYNPSSRATAEEALRHEWFRTEPKPGPNVFRM-------------------
H. sapiens (CDK8)      ----------------KPDSKAFHLLQKLLTMDPIKRITSEQAMQDPYFLEDPLPTSDVFAGCQ-----IPYPKREFLTEE
S. cerevisiae (SRB10)  -----------------RDKHALSLLYHLLNYDPIKRIDAFNALEHKYFTESDIPVSENVFEGL----TYKYPARRIHTND

A. thaliana            DFEGTTSINPPQA---------------------------------VAAGNVAGNMAGAHGM-----GSRSM-PRPMVAHN
O. sativa#1            DFEGTTSLQPTQA---------------------------------PSGNAAPGNQSVVPRP-----IPRQM-QQP--MVG
A. trichopoda#1        DFEGSTSVQASQPV--------------------------------SSGNAVSGSLAAPSVV-----AARPV-PRQMPMVG
A. trichopoda#2        DFEGSTSVQASQP--------------------------------------------------------------------
O. sativa#2            AFNQL----------------------------------------------------------------------------
P. engelmanii          DFDGTITTQPSQV---------------------------------SSGNAPSGSMAAAAVA-----AVRPI-PQQMQLMG
S. moellendorffii      DFEGSGSIQNTQMVRNRKRKQAFFSPLQCGGGSSFLMVSTFRGSQMSTSNSLPASAASAAAASATANAVRPMHHQQMPLVG
M. polymorpha          DFDGSTAPPQS-----------------------------------NTNLAPQQQQRGEHMM-----QVPAM-HQPLHLPA
P. patens              DFDGTTAPPPSQM---------------------------------PNSSNMSGSSNQLHPS-----MNVNM-QQQ-----
K. nitens              DMGSN---EPTPV---------------------------------GNTANPASGQQ------------------------
Coccomyxa sp. Obi      ---GASMPPGAPL---------------------------------NTFDAPQARLGP----------SRNA-TPTAVLGP
C. reinhardtii         VPAGLPGAAGAGG---------------------------------GPGDGGGGGGLPPPPG--PPGGGRAG-GGPGGPGG
Volvox carteri         -------QVGSWN---------------------------------GNGHGPGGSCWA-----------------------
H. sapiens (CDK8)      EPDDKGDKKNQQQ---------------------------------QQGNNHTNGTGH-------PGNQDSS---------
S. cerevisiae (SRB10)  ---NDIMNLGSRT---------------------------------KNNTQASGITAGAAA------------NALGGLGV
                *  .

A. thaliana            MQRMQQSQGMMAYNFPAQ---AGLNP--SVPLQQQRGM--AQPHQQQQLRRKDPGMGM---SGYAPPNKSRR--------L
O. sativa#1            MSRMGGTN-MAAFGAAPQGGIAGMNP-GNIPMQRGAG---AQSHPHQLRRKADQGMGMQN-PGY-PTQQKRR--------F
A. trichopoda#1        MPRMPGAPGIAAFNLSSQASMAGLNP-NNIPMPRA-----ASQAHQQQLRRKDPGMGMPN-TGY-PQQKSRR--------F
A. trichopoda#2        -----------------------------------------SRYLQ----------GMQF-QGV-WQLLL-----------
O. sativa#2            -KRHQGTQ-LLATSLWYRDPFRGKCN-NPWSVCREWVV-----------------------QTWRPLVQLRKE-------A
P. engelmanii          MQRMQNAN-MAAFNLGAQASMSGLNH-NNLPLQRGSS---QQQAHQQQVRRKEPNSGFPN-PGYPPQPKSRR--------L
S. moellendorffii      MQRLQTAS-MGAFNVGAQPAMAGLAA--NMFVQRGQQL--QQVAHHQQQQQQQQQQQHQHHQHQQHQVQKLRSF----LRS
M. polymorpha          IQRMPQNN-MNNFS-----------------------------SVNQQGRRKEPDSGNQNYNNYQPLNKSRR--------M
P. patens              -HRIQNSN-TLSVN--QPVGMHGMNSSSNMYIQRGNATVPQQQPQQQQGLRRKDQTNVGYPGHYQPPNKSRR--------I
K. nitens              -RRFQQQG--------------GDQSGSG---QGGTTTG-SRLGPGGSGRRSDGRS-----QGQGPSKMQRR---------
Coccomyxa sp. Obi      DRRLTPVN-AAVTTHTTP---QGLPPGSA--------------------------------SRPRPPLKRPHQD--P-WLR
C. reinhardtii         GMRGSSQA-AAML------------------------------------------------AQSGPQGKRRRMENVP-GFR
Volvox carteri         ---------------------------------------------------------------------------------
H. sapiens (CDK8)      -------------------------------------------------------HTQGPPLKKVRVVPPTTTSGGLIMTS
S. cerevisiae (SRB10)  NRRILAAA-AAAAA-----AVSGNN------------------------------------ASDEPSRKKNR--------R
```

Supplementary figure 3: Multiple sequence Alignment of CDKE proteins from different plant groups along with Human (CDK8) and Yeast (SRB10). Conserved motifs are marked in different colour schemes.

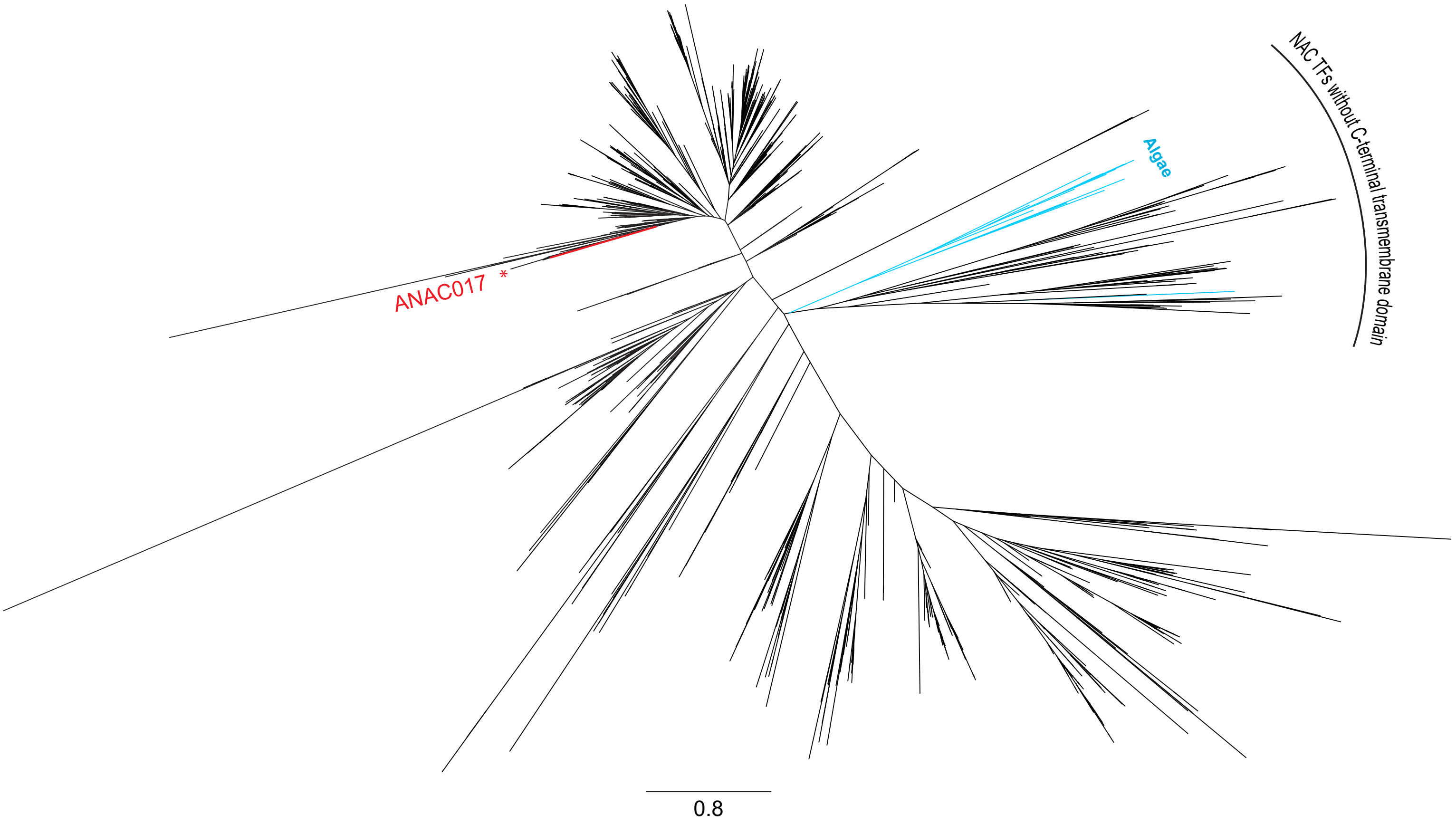

Supplementary Figure 4: **Phylogenetic analysis of ANAC017/transmembrane domain containing NACs in Archaeplastida**

NAC sequences from different groups of plants (Archaeplastida) were obtained from the One KP database by using ANAC017 (containing C-terminal transmembrane domain) as query sequence in the protein blast tool. A total of 880 NAC sequences representing algae, bryophytes, lycophytes, ferns, gymnosperms, and angiosperms, were obtained from the One KP database. The obtained sequences, along with Arabidopsis NACs obtained from PLAZA, were aligned by MAFFT and the IQ-tree was constructed with 1000 bootstraps. Figtree was used to annotate the phylogenetic tree. NAC TFs from land plants are shown in black colour, while cyan colour was used to show NAC TFs present in algae. Arabidopsis ANAC017 is also indicated in red colour with an asterisk. Additionally, NAC TFs that lack the C-terminal transmembrane domain (identified in our BLAST search results) are also marked in the tree.
